# Low-Temperature Sulfidic-Ice Microbial Communities, Borup Fiord Pass, Canadian High Arctic

**DOI:** 10.3389/fmicb.2018.01622

**Published:** 2018-07-24

**Authors:** Christopher B. Trivedi, Graham E. Lau, Stephen E. Grasby, Alexis S. Templeton, John R. Spear

**Affiliations:** ^1^Department of Civil and Environmental Engineering, Colorado School of Mines, Golden, CO, United States; ^2^Department of Geological Sciences, University of Colorado Boulder, Boulder, CO, United States; ^3^Geological Survey of Canada, Calgary, AB, Canada

**Keywords:** sulfur, microbial ecology, Canadian Arctic, glacier, Europa, astrobiology, low-temperature

## Abstract

A sulfur-dominated supraglacial spring system found at Borup Fiord Pass (BFP), Ellesmere Island, Nunavut, Canada, is a unique sulfur-on-ice system expressed along the toe of a glacier. BFP has an intermittent flowing, subsurface-derived, glacial spring that creates a large white-yellow icing (aufeis) that extends down-valley. Over field campaigns in 2014, 2016, and 2017, numerous samples were collected and analyzed for both microbial community composition and aqueous geochemistry. Samples were collected from multiple site types: spring discharge fluid, aufeis (spring-derived ice), melt pools with sedimented cryoconite material, and mineral precipitate scrapings, to probe how microbial communities differed between site types in a dynamic freeze/thaw sulfur-rich system. Dissolved sulfate varied between 0.07 and 11.6 mM and was correlated with chloride concentrations, where the fluids were saltiest among spring fluids. The highest sulfate samples exhibited high dissolved sulfide values between 0.22 and 2.25 mM. 16S rRNA gene sequencing from melt pool and aufeis samples from the 2014 campaign were highly abundant in operational taxonomic units (OTUs) closely related to sulfur-oxidizing microorganisms (SOM; *Sulfurimonas, Sulfurovum*, and *Sulfuricurvum*). Subsequent sampling 2 weeks later had fewer SOMs and showed an increased abundance of the genus *Flavobacterium*. *Desulfocapsa*, an organism that specializes in the disproportionation of inorganic sulfur compounds was also found. Samples from 2016 and 2017 revealed that microorganisms present were highly similar in community composition to 2014 samples, primarily echoed by the continued presence of *Flavobacterium* sp. Results suggest that while there may be acute events where sulfur cycling organisms dominate, a basal community structure appears to dominate over time and site type. These results further enhance our knowledge of low-temperature sulfur systems on Earth, and help to guide the search for potential life on extraterrestrial worlds, such as Europa, where similar low-temperature sulfur-rich conditions may exist.

## Introduction

Microorganisms that use sulfur as a source of energy are ubiquitous on Earth and are found in diverse environments (Dahl and Friedrich, 2008; Klotz et al., 2011). In marine hydrothermal fields there are an abundance of organisms spanning the Proteobacteria that are involved in sulfur redox reactions (Perner et al., 2007). Sulfur oxidizing organisms are also found in deep cave systems, where chemolithoautotrophy is utilized to oxidize reduced sulfur species as electron donors creating biofilms that contain up to 50% S^0^ by mass (Hamilton et al., 2015). Due to its ability to serve as both an electron donor and/or electron acceptor across an eight electron transfer redox state (−2 to +6), sulfur is a primary ingredient for life on Earth, and in particular to microorganisms across a multitude of environments.

One of these extreme environments is the sulfur-dominated supraglacial spring system found at Borup Fiord Pass (BFP), Ellesmere Island, Nunavut, Canada (Grasby et al., 2003). BFP is one of the few known low-temperature springs in the Canadian Arctic, along with Gypsum Hill, Colour Peak on Axel Heiberg Island, and Ice River on Ellesmere Island (Pollard et al., 1999; Grasby, 2003; Perreault et al., 2007; Niederberger et al., 2009; Gleeson et al., 2011; Grasby et al., 2012; Wright et al., 2013; Lau et al., 2017). The spring at BFP is unique as it discharges from a glacier and has very high concentrations of dissolved hydrogen sulfide (H_2_S) for a geological system (Sievert et al., 2007; Gilhooly et al., 2014), the highest of which was measured from spring fluid collected in 2009 at 3.99 mM. Biotic and/or abiotic oxidation of H_2_S to elemental sulfur creates visually striking yellow glacial ice, as well as yellow colored aufeis formed by spring discharge into sub-zero air temperatures. This sulfur-covered ice extends for tens to thousands of square meters down valley (Grasby et al., 2003; Gleeson et al., 2012; Lau et al., 2017). Spring water discharging from the glacier is briny, sulfide-rich, and hosts a diverse microbial community that may make use of sulfur metabolisms for growth (Grasby et al., 2003; Gleeson et al., 2011; Wright et al., 2013). When discharging during cold winter months the spring water freezes, forming sulfidic aufeis as well as associated cryogenic minerals (Grasby, 2003; Grasby et al., 2003; Lau et al., 2017). During the subsequent summer months surface melt pools form, releasing trapped hydrogen sulfide gas that is then oxidized to elemental sulfur that accumulates as sulfur-rich cryogenic materials (Lau et al., 2017). It remains unclear to what degree, H_2_S oxidation is driven by microbial interaction (Grasby et al., 2003; Gleeson et al., 2011; Wright et al., 2013), and how microbial community dynamics function in these complex sulfidic-ice environments.

The aim of this study was to use 16S rRNA gene sequencing coupled to geochemical analysis to identify and better constrain the microbial consortia that have adapted to live in this sulfur-rich, low-temperature environment. To address this, field campaigns to BFP over multiple years were undertaken to collect samples from multiple site types (spring discharge, aufeis, mineral precipitates, and melt pool waters).

### BFP site background

**Figure 1:**
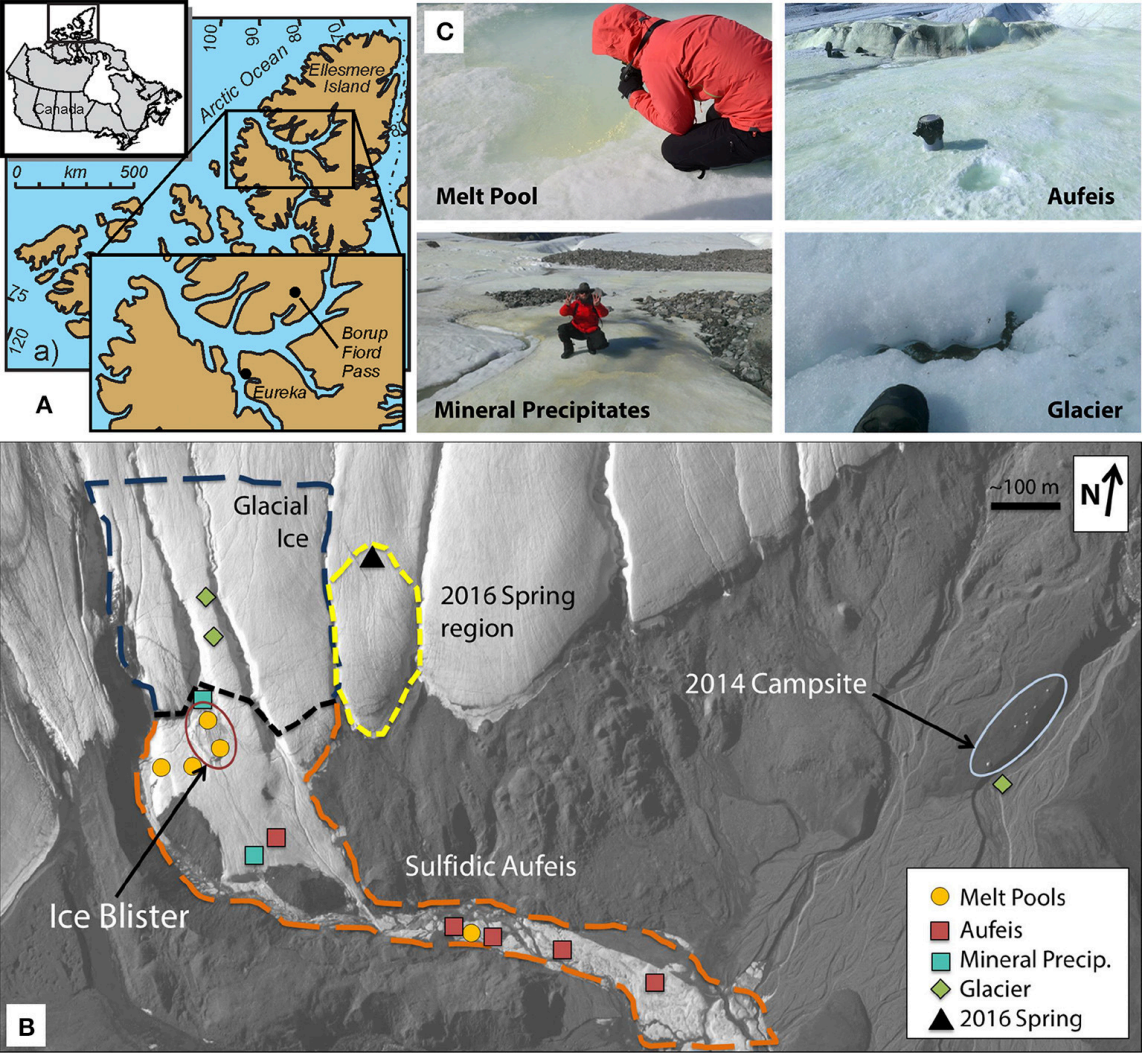
Borup Fiord Pass: Sample locations and types. **(A)** Line drawing and map showing the location of Borup Fiord Pass in the Canadian High Arctic. The inset shows the location of Borup Fiord Pass on Ellesmere Island (from Grasby et al., 2012). **(B)** Satellite imagery of the toe of the coalescence glacier taken 2 July, 2014 during this sampling campaign. Highlighted sample sites are melt pool (gold circles), aufeis (red squares), mineral precipitates (teal square), and glacier samples (green diamonds). The location of the previous 2016 spring (black triangle) is also shown. Also highlighted are general regions: Glacial Ice (dark blue dashed line), Sulfidic Aufeis (orange dashed line), the area referred to as the Ice Blister (red oval), as well as the region of a newly emerged spring from 2016 (yellow dashed line). 2017 mineral precipitate samples are not shown, however, samples were collected within the Sulfidic Aufeis zone near the Ice Blister from 2014 (this can be seen in Figure S3a). Also note the location of the 2014 campsite on the far east side of the figure for added scale. **(C)** Examples of melt pool (top left), aufeis (top right), mineral precipitate (bottom left), and glacier (bottom right) sample types. Professor Alexis Templeton (top left), a five gallon rock pail (top right), Dr. Graham Lau (bottom left), and a hiking boot (bottom right) for relative scale. *Satellite image courtesy of the DigitalGlobe Foundation*.

Previous research at BFP includes data about spring geochemistry, permafrost-hydrogeology, microbiology, biomineralization, and putative sulfur metabolisms (Grasby et al., 2003, 2012; Gleeson et al., 2010, 2011, 2012; Scheidegger et al., 2012; Wright et al., 2013; Lau et al., 2017). BFP is at the height of a north-south trending valley through the Krieger mountains at 81° 01′ N, 81° 38′ W (Figure 1A). The spring system is approximately 210–240 m above sea level and discharges from glacial ice near the toe of two coalesced glaciers (Grasby et al., 2003). The spring system is thought to be perennial but discharges from different locations of the glacier from year to year (Grasby et al., 2003; Gleeson et al., 2010; Wright et al., 2013; Lau et al., 2017). Given the lack of winter observations due to 24-h darkness, it cannot be stated with certainty that the spring is perennial; however, the annual formation of extensive sulfidic aufeis in the proglacial area indicates active discharge during the winter. The aufeis formed is characterized by extensive coverage of elemental sulfur, calcite, and gypsum, as well as rare minerals of vaterite and β- and γ-cyclooctasulfur (Grasby, 2003; Grasby et al., 2003; Lau et al., 2017), all related to cryogenic processes. It is not clear, however, in what way microorganisms may play a part in their formation or utilization. A lateral fault approximately 100 m south of the toe of the glacier has been implicated in playing a role in the subsurface hydrology, in that this may be one of the areas where subsurface fluids are able to contact the surface (Grasby et al., 2003; Scheidegger et al., 2012). Furthermore, it has been suggested that this fault may facilitate fluid flow underneath the glacier, which could deliver available organic carbon for potential subsurface microbial processes such as sulfate reduction (Grasby et al., 2003). This process would generate sulfide, which travels to the surface as dissolved aqueous phase sulfide for abiotic surface oxidation and/or biological metabolism.

## Materials and methods

### Field work and sample collection

Sample material for this study was collected during a 2-week period in 2014 (21 June thru 2 July) as well as during single-day visits in 2016 (4 July) and 2017 (7 July). Samples included: (1) background control samples of glacial fluid (G1-G3); (2) fluid from an active sulfide-spring in 2016 (Spring 2016); (3) melted and filtered fluid from the aufeis (A1–A6); (4) fluid and cryoconite material collected from the melt pools on the surface of the aufeis (M1–M6); and (5) mineral precipitates that were scraped from the surface of the aufeis (two from 2014 and five from 2017; AS1–AS7).

Cryoconite sediments (melt pools and sample G1) were collected using sterile and field-washed transfer pipettes. Up to 0.5 g of this material plus water was placed into ZR BashingBead™ lysis tubes (Zymo Research Corp., Irvine, CA, USA) containing 750 μL of Xpedition™ Lysis/Stabilization Solution (Zymo Research Corp.) and homogenized for 45 s using the Zymo Xpedition™ portable impact drill (Zymo Research Corp.). Samples were then stored on ice until DNA extraction was performed. Mineral precipitate samples from 2014 (AS1 & AS2) were collected in 50 mL polypropylene conical tubes (VWR International, Radnor, PA, USA). Samples were scraped from surface ice, and the resulting mixture was melted then filtered through 0.22 μm Luer-lok Sterivex™ filters (EMD Millipore; Darmstadt, Germany), which were then capped and kept on ice in the field until returning to the laboratory where they were stored at −20°C until extraction. Mineral precipitate scrapings from the 2017 field campaign (AS3–7) were collected using sterile and field-washed transfer pipettes and up to 0.5 g of material were mixed in ZR BashingBead™ lysis tubes containing 750 mL of DNA/RNA Shield™ (Zymo Research Corp.). Samples were shaken and kept at 4°C until returned to the laboratory, where they were then stored at −80°C until DNA/RNA extraction could be performed. Aufeis samples were prepared by scraping approximately 10 cm of surface ice away and then digging out an area approximately half a meter wide, 30 cm tall, and 15 cm deep. The ice was placed in field-washed five gallon pails and allowed to thaw. Spring water fluid (Spring 2016) and melted aufeis (A1–A6) samples were prepped for DNA extraction by filtration through 0.22 μm Luer-lok Sterivex™ filters (EMD Millipore), which were then capped and kept on ice in the field until returning to the laboratory where they were stored at −20°C until extraction. Filtered water volumes are shown in Table S3. Fluids for aqueous geochemical measurement were filtered with 0.22 μm polyethersulfone (PES; EMD Millipore, Billerica, MA, USA) filters into 15 mL polypropylene tubes for ion chromatography (IC) and inductively-coupled plasma optical emission spectroscopy (ICP-OES) for measurement of major anions and cations, respectively. Samples for ICP-OES were acidified with approximately 0.1 mL nitric acid in the field.

### DNA extraction and 16S rRNA gene library sequencing

DNA extraction of 2014 cryoconite (melt pool and glacial ice) samples was performed using the Xpedition™ Soil/Fecal DNA MiniPrep kit (Zymo Research Corp.). DNA extraction of fluids collected via 0.22 μm Luer-lok Sterivex™ filters (melt pool, spring discharge, aufeis, and 2014 mineral precipitates) was performed using the MO BIO PowerWater® Sterivex™ DNA Isolation Kit (QIAGEN, Hilden, North Rhine-Westphalia, Germany), and DNA of 2017 mineral precipitate samples was extracted using the ZymoBIOMICS™ DNA/RNA Mini Kit (Zymo Research Corp.). All extractions were performed according to the manufacturer's instructions. Extracted DNA was amplified via PCR using 16S rRNA V4 primers 515F_Y and 926R (Parada et al., 2016). The Forward primer (M13-515_Y: 5′-**GTA AAA CGA CGG CCA GT**C CGT GYC AGC MGC CGC GGT AA-3′) contains the M13 forward primer (bold) ligated to the 16S rRNA gene-specific sequence (underlined) to allow for barcoding in a subsequent PCR reaction (Stamps et al., 2016). The reverse primer (926R: 5′-CCG YCA ATT YMT TTR AGT TT-3′) was described in Parada et al. (2016).

Each 25 μL PCR reaction mixture consisted of: 1X 5PRIME HOT MasterMix (Quantabio, Beverly, MA, USA), 0.2 μM of each primer, molecular grade water, and 2 μL of extracted template DNA. Positive (ZymoBIOMICS™ Microbial Community Standard; Zymo Research Corp.) and negative (no template) controls were also amplified along with sample template reactions. PCR included the following parameters: initial denaturation at 94°C for 2:00 min, 30 cycles of (94°C for 45 s, 50°C for 45 s, and 68°C for 1:30 min), final extension of 68°C for 5 min, and a final 4°C hold. A second, six cycle PCR was used to add a unique 12 bp barcode (Hamady et al., 2008) to each previously amplified sample using a forward primer containing the barcode+M13 forward sequence (5′-3′) and the 926R primer (see mapping file, Table S1). The final barcoded PCR products were cleaned using KAPA Pure Beads (KAPA Biosystems Inc., Wilmington, MA, USA) at a final concentration of 0.8X v/v, quantified using the Qubit® dsDNA HS assay (Life Technologies, Carlsbad, CA, USA), pooled in equimolar amounts, and concentrated to a final volume of 80 μL using two Amicon® Ultra-0.5 mL 30K Centrifugal Filters (EMD Millipore, Billerica, MA, USA).

The final pooled library was run on the Illumina MiSeq platform (Illumina, San Diego, CA, USA) at the Duke Center for Genomic and Computational Biology (Duke University, Durham, NC, USA) using PE250 V2 chemistry. After sequencing, reads were merged and de-multiplexed using QIIME (Caporaso et al., 2010b), filtered by quality, clustered into operational taxonomic units (OTUs) at 97% identity, and chimera checked using VSEARCH (Rognes et al., 2016). OTU taxonomy was assigned using UCLUST (Edgar, 2010) and the SILVA database (Release 123; Pruesse et al., 2007). Representative sequences were aligned using pyNAST (Caporaso et al., 2010a) against an aligned version of the SILVA r123 database. A mapping file is included as Table S1, and the commands used to produce the final BIOM file are publicly available at https://doi.org/10.5281/zenodo.1207309. Additional diversity metrics including alpha rarefaction by observed OTUs, rank abundance, and sequencing coverage are included in the supplemental material (Figures S5, S6, and Table S2, respectively).

Estimated relative biomass for each sample was calculated based on sample amount and extracted DNA quantity as measured on the Qubit® fluorometer (Life Technologies, Carlsbad, CA, USA). DNA concentration was multiplied by the elution volume from extraction (to get ng of DNA) and then another ~53% was added by weight to account for the mass lost during extraction. This percentage (53%) was calculated based on the volume of starting lysis buffer (750 μl) prior to bead beating divided by the volume that is taken through the rest of the protocol (400 μl). Total estimated biomass of a sample (cells mL^−1^ or cells mg^−1^, depending if the sample was extracted from a filter or sediment, respectively) was calculated based on an empirical value of 2.5 fg DNA cell^−1^ (Button and Robertson, 2001) from aquatic microorganisms.

### Aqueous geochemistry

Fluid pH (for 2014 and 2017 samples) was measured in the field with test strips (Carolina Biological Supply Co.; Burlington, NC, USA). Temperatures were measured with a Hach HQ40d portable field meter (Hach Company, Loveland, CO, USA). For 2014 samples, ICP-OES and IC measurements were performed on an ARL (Applied Research Laboratories) 3410+ ICP-OES and a Dionex (Thermo Scientific; Waltham, MA, USA) 4500I IC, respectively, at the Laboratory for Environmental and Geological Studies (LEGS) at the University of Colorado Boulder. Total organic carbon (TOC) measurements for cryoconite material from M1 were conducted according to Lau et al. (2017).

Spring fluid collected during the summer of 2016 was analyzed at the Colorado School of Mines. Major anions were measured using a Dionex (Thermo Scientific; Waltham, MA, USA) ICS-90 ion chromatography system running an AS14A (4 × 250 mm) column. Major cations were measured using a Perkin-Elmer (Waltham, MA, USA) Optima 5300 DV ICP-OES. BFP-2016 spring fluid was filtered and acidified in the same fashion as above for 2014 samples. Due to low/no-flow conditions, aqueous geochemistry data for 2017 samples was not collected.

**Figure 2:**
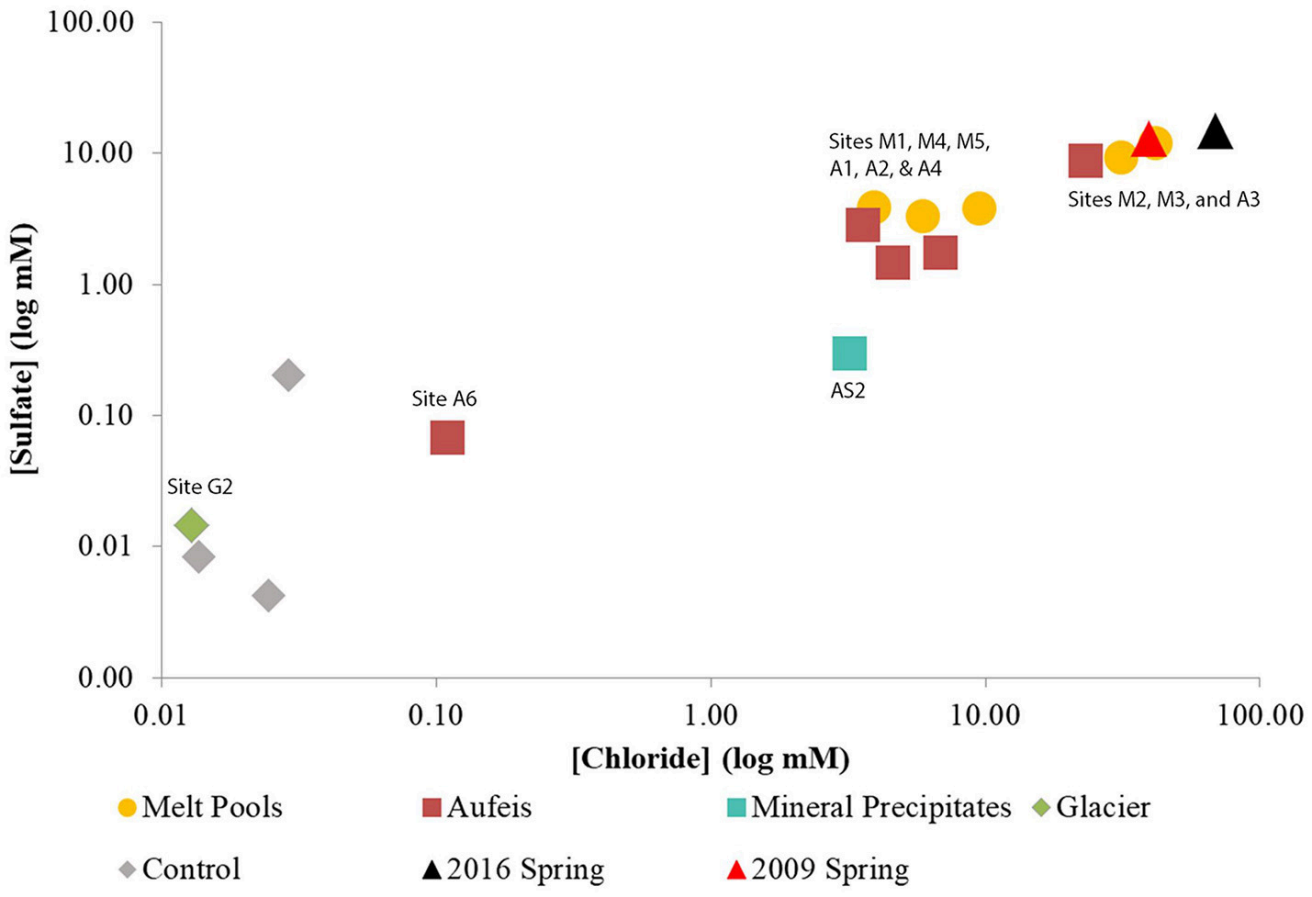
Log-log plot of sulfate vs. chloride aqueous geochemistry data. Sulfate and chloride aqueous geochemistry data appear to trend together. Note the “Control” samples which have been added for geochemical comparison, however, there is no 16S rRNA gene sequencing data associated with these samples. Both sites (M4 and M5) which were located on top of the Ice Blister are lower in sulfate and chloride concentrations than those located just to the west (Sites M2 & M3) and south (G2). These also match closely with aqueous geochemistry data taken from spring fluids from 2009 to 2016.

Aqueous geochemistry was ordinated in R v3.4.3 (R Core Team, 2016) using the community ecology package “vegan” (Oksanen et al., 2016). Data was ordinated using the built-in Hellinger standardization. A principal coordinates analysis (PCoA) plot was created using the packages “ggplot2” and “ggfortify” (Tang et al., 2016; Wickham, 2016) in order to determine which geochemical factors might influence observed differences among the different site types (Figure 2 and Figure S4).

## Results

### General field observations

The 2014 field season at BFP revealed a large sulfur-covered area of ice, Sulfidic Aufeis (Figure 1B). This area included an approximately 25 m diameter, sulfur-rich mound (herein referred to as the Ice Blister) at the toe of the glacier as well as what was likely spring-derived ice that was approximately 5 × 10^4^ m^2^ in size and approximately 5 m thick at greatest extent (Lau et al., 2017). The majority of the aufeis had built up around the toe of the glacier while the rest followed a channel southeast down the valley for approximately 2 km (Figure 1B). While no flowing spring was observed during 2014, it is thought that this spring-derived aufeis likely originated from within the sulfidic zone of the Ice Blister (Figure 1B). Ice blister formation (also known as a frost mound) is a common feature that forms in polar zones when fluid flow is present, often in the form of a spring (Pollard, 2005; Wainstein et al., 2008), and it was likely that the spring was active sometime prior to arrival at the site in 2014.

The locations of sampled melt pools, aufeis sites, mineral precipitate sites, glacier sites, and the Ice Blister are shown in Figure 1B, which is a satellite image captured on 2 July 2014. For comparison, the location of the 2016 spring discharge site is labeled to the east of the Sulfidic Aufeis zone from 2014. Mineral precipitate samples were all collected within the Sulfidic Aufeis zone, closer to the Ice Blister (see Figure S3). These samples were chosen due to their variability in material type (i.e., fluid, ice, precipitate, etc.) as well their potential to represent how microbial communities may or may not change in a dynamic low-temperature environment.

### Estimated biomass and sample QC

Estimated biomass values (calculated from extracted DNA; Table S3) across all samples averaged 1.30 × 10^4^ cells mL^−1^ and 1.20 × 10^5^ cells mg^−1^, depending on sample type (fluid or solid material). Average biomass calculated for each site type were as follows: 2.63 × 10^3^ cells mL^−1^ and 4.34 × 10^3^ cells mg^−1^ for melt pools; 2.70 × 10^3^ cells mL^−1^ for aufeis; 6.36 × 10^4^ cells mg^−1^ and 1.55 × 10^5^ cells mL^−1^ for mineral precipitate samples; and 7.65 × 10^4^ cells mg^−1^ and 1.29 × 10^4^ cells mL^−1^ for glacier samples. The 2016 spring fluid sample had a calculated biomass of 3.11 × 10^3^ cells mL^−1^. It should be noted that biomass values are most likely an underestimate due to potential loss of DNA during extraction.

### Melt pools and 2016 spring fluid samples

Along with the Ice Blister feature, melt pools (top left panel, Figure 1C) were observed across the aufeis surface (see Figure S1). Melt pools (comprising samples M1-M6) close to the Ice Blister exhibited a strong odor of hydrogen sulfide and had a yellow opaque layer that had formed across the surface of the water. Melt pool sizes varied but averaged 1–2 m in width and length and were between 10 and 20 cm in depth. Many of the melt pools had cryoconite material on the bottom that was white/yellow in color and in at least one, was found to contain elemental sulfur and other sulfate minerals (Lau et al., 2017). Sites M4 and M5 are located on the south and north ends of the Ice Blister, respectively, while M2 and M3 are just to the west of the Ice Blister (Figure S1). Sites M1 and M6 are the furthest removed from the Ice Blister, one being at the bottom of a flowing waterfall (M6) and the other being the furthest most melt pool sampled down the Sulfidic Aufeis (M1). The spring discharge sample from 2016 was to the east of the 2014 Sulfidic Aufeis and emanated higher up on the glacier (Figure 1B). The 2016 spring fluid is grouped with the melt pool samples based on similar community resemblance and aqueous geochemistry. Sulfide values for melt pool samples were measured for site M2 and from ice near to sites M3 and M4 (0.220, 1.00, and 2.25 mM respectively; Table 1). Sulfide was not measured for the 2016 spring sample. Sulfate values were measured for melt pool samples at sites M1-M5, and ranged in value from 3.28 to 11.6 mM. The 2016 spring sample contained 14.8 mM sulfate, which is the highest reported value in this study. Chloride values for melt pools and the 2016 spring ranged from 3.96 to 68.79 mM. The latter value belongs to the 2016 spring, again, the highest reported chloride value of this study.

**Table 1:** Aqueous geochemistry for selected BFP sites.

	**M1**	**M2**	**M3**	**M4**	**M5**	**A1**	**A2**	**A3**	**A4**	**A6**	**AS2**	**AS3^b^**	**AS4^b^**	**G2**	**2016 Spring**	**2009 Spring**
**Date**	**Jun 21**	**Jun 21**	**Jun 26**	**Jun 27**	**Jun 30**	**Jun 25**	**Jun 25**	**Jun 28**	**Jun 28**	**Jun 30**	**24-Jun**	**Jul 7 17**	**Jul 7 17**	**Jun 30**	**Jul 4 16**	**Jul 09**
**Type**			**Melt pool**					**Aufeis**				**Mineral precipitate**		**Glacier**	**Spring fluid**	
Sulfide^a^	(NM)	0.2200	1.0000	2.25	(NM)	0.5200	(NM)	(NM)	0.3200	(NM)	(NM)	(NM)	(NM)	(NM)	(NM)	3.9900
F^−^	0.0127	0.0326	0.0266	(DL)	0.0128	0.0116	(DL)	0.0248	(DL)	(DL)	(DL)	(NM)	(NM)	(DL)	0.0426	0.0542
Cl^−^	9.5815	41.9671	31.5257	3.9628	5.9468	6.8968	4.6456	23.3210	3.6215	0.1105	0.29126	(NM)	(NM)	0.0128	68.7924	39.2000
Br^−^	(DL)	(DL)	0.0093	(DL)	(DL)	(DL)	(DL)	(DL)	(DL)	(DL)	(DL)	(NM)	(NM)	(DL)	0.0312	0.0330
${\text{NO}}_{3}^{-}$	(DL)	(DL)	(DL)	(DL)	(DL)	(DL)	(DL)	(DL)	(DL)	(DL)	(DL)	(NM)	(NM)	0.0189	(DL)	0.0030
${\text{SO}}_{4}^{2-}$	3.7149	11.6948	9.1208	3.8064	3.2843	1.7004	1.4300	8.6216	2.8018	0.0666	3.22504	(NM)	(NM)	0.0146	14.8151	13.0000
S_2_${\text{O}}_{3}^{2-}$	(DL)	0.2182	(DL)	(DL)	(DL)	(DL)	(DL)	(DL)	(DL)	(DL)	(DL)	(NM)	(NM)	(DL)	(DL)	0.1030
Si	0.0224	0.0940	0.0477	0.0096	0.0050	0.0075	0.0039	0.0459	0.0050	(DL)	3.14028	(NM)	(NM)	(DL)	0.0887	0.0411
Mn_T_	0.0002	0.0004	0.0004	0.0002	(DL)	0.0002	0.0002	(DL)	(DL)	0.0004	0.0032	(NM)	(NM)	(DL)	0.0007	0.0010
Fe_T_	(DL)	(DL)	(DL)	(DL)	0.0005	(DL)	(DL)	0.0004	0.0002	(DL)	(DL)	(NM)	(NM)	(DL)	0.0000	0.0050
Mg^+^	1.5626	6.9665	3.9193	0.7427	0.8171	1.1779	0.6223	3.3213	0.8261	0.0177	0.07069	(NM)	(NM)	(DL)	10.1510	12.9000
Ca^2+^	3.8263	11.3045	9.3785	3.5040	2.8575	1.7765	1.4410	8.2013	2.7488	0.1085	3.2365	(NM)	(NM)	0.0053	14.7511	17.3000
Na^+^	6.0439	27.0679	15.9823	2.8462	3.0520	4.3296	2.1968	13.6151	3.2334	0.1061	0.29406	(NM)	(NM)	(DL)	39.6291	51.8000
K^+^	0.0470	0.1991	0.0991	(DL)	(DL)	0.0414	0.0239	0.0889	0.0128	0.0128	0.01049	(NM)	(NM)	0.0320	0.2619	0.3020
pH	5.5–6.8	5.5-6.0	(NM)	(NM)	(NM)	(NM)	(NM)	(NM)	(NM)	(NM)	(NM)	7.0	7.0	(NM)	(NM)	(NM)

*Aqueous geochemistry values (in mM) for the selected melt pool, aufeis, mineral precipitate, glacier, 2016 and 2009 spring fluids. Also presented are the category type and the date each sample was collected. Aqueous geochemistry values are those used to calculate the charge balance error, which is presented in Table S3 along with microbiological data. Values presented as (DL) were below the detection level of the instrument.Values presented as (NM) were not measured for that geochemical parameter. Relevant DL values are 1.1 μM (F^−^), 0.6 μM (Br^−^), 0.8 μM (${\text{NO}}_{\text{3}}^{-}$), 1.8 μM (S_2_${\text{O}}_{\text{3}}^{\text{2-}}$), 0.7 μM (Si), 0.02 μM (Mn_T_), 0.1 μM (Fe_T_), 0.6 μM (Mg^+^), 1.9 μM (Na^+^), 4.0 μM (K^+^)*.

a *All sulfide data were collected on 28 June 2014*.

b *Only pH was collected for samples during 2017*.

**Figure 3:**
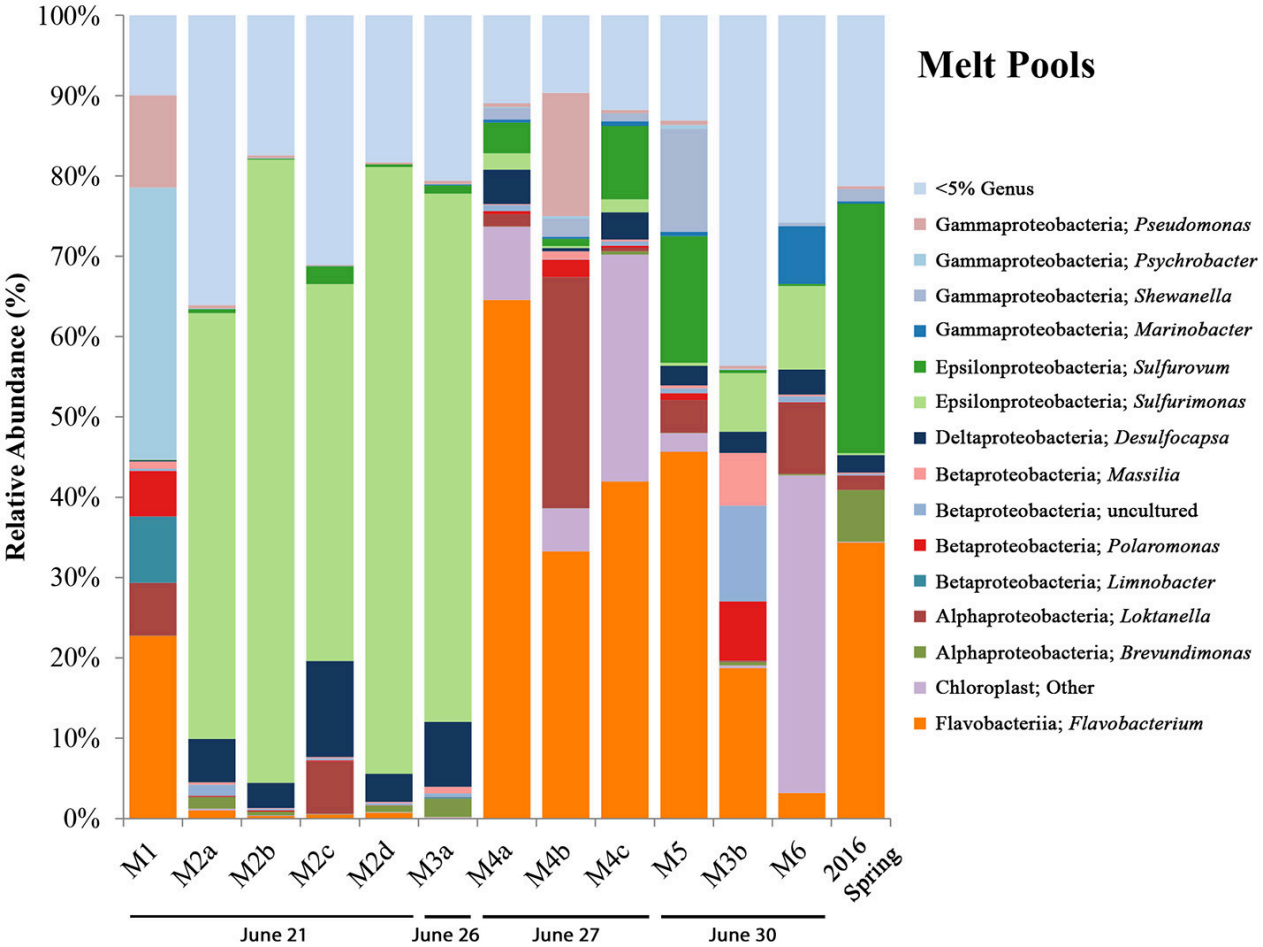
BFP melt pool bar charts. Bar charts show relative abundance of 16S rRNA gene sequencing libraries, where only OTUs representing organisms more than 5% of any given sample are labeled. All other OTUs below 5% relative abundance are grouped together in the category “Genus <5%.” Labels show Class and subsequent Genus level. Sites are in order of sampling date from left to right. All melt pool sites are within the Sulfidic Aufeis zone. Sites are named such that any replicates are denoted by lowercase letters. Exact locations of all melt pool sites can be seen in Figure S1.

Initial inquiry into the microbial communities of melt pool sites shows a wide range of organisms among the phylum Proteobacteria (Figure 3). Many of the operational taxonomic units (OTUs) most closely related to known genera (e.g., *Pseudomonas, Psychrobacter, Shewanella, Marinobacter, Loktanella, Massilia, Polaromonas*, and *Limnobacter*) are ones that have been found in polar and glacial environments previously (Van Trappen, 2004; Bej et al., 2009; González-Toril et al., 2009; Gleeson et al., 2011; Zhou et al., 2015). An OTU most closely related to the genus *Flavobacterium* was highly abundant in a number of melt pool samples (M1, M3b, M4a-c, and M5). When queried using NCBI BLAST, the top hit (99% identity) was most closely related to *Flavobacterium psychrolimnae* (GenBank Accession: KY047392.1) discovered in a wetland sample. This appears to be a common theme in a majority of samples collected at BFP, where *Flavobacterium* was a dominant organism across all sample types and across different years.

Regarding sulfur cycling in the Epsilonproteobacteria, OTUs most closely related to the sulfur-oxidizing microorganisms (SOMs) *Sulfurovum* and *Sulfurimonas* are also present, and have been previously observed at BFP (Gleeson et al., 2011; Wright et al., 2013). Site M1, located southeast on the Sulfidic Aufeis, was dominated by genera commonly found in polar environments, such as *Pseudomonas, Psychrobacter, Polaromonas, Limnobacter*, and *Loktanella* (11.5, 33.8, 5.7, 8.2, and 6.6%, respectively). Site M2 was dominated by an OTU most closely related to the genus *Sulfurimonas*, across all four replicates (a–d), all greater than 45% relative abundance. This is also true for biological replicate M3a, but not for M3b, potentially due to the different sampling date. An OTU most closely related to *Sulfurovum* was found in small amounts in site M2 (<5%) and larger amounts in sites M4 (3.8–9.1%), M5 (15.7%), and the 2016 Spring fluid (31.1%). The presence of an OTU most closely related to the Deltaproteobacterial genus *Desulfocapsa* was also found in many of the melt pool sites, with the highest abundance being seen in M2 and M3, at 11.9 and 8.1% respectively. *Desulfocapsa* is a relatively well-known sulfur-disproportionating microorganism which has been found mostly in subsurface sediments (Finster et al., 1998; Zeng et al., 2017), but was also shown to be present in Antarctica's “Blood Falls” (Mikucki and Priscu, 2007).

An OTU most closely related to the Alphaproteobacterial genus *Brevundimonas* was present in low abundance in sites M2 and M3, with the largest abundance seen in the 2016 spring sample at 6.4%. This organism was also found previously in Antarctic and alpine environments (González-Toril et al., 2009) and was implicated as psychrotolerant and highly radiation resistant (Dartnell et al., 2010). Also of note were appreciable amounts of an OTU most closely related to chloroplast, especially in sites M4 (>5%) and M6 (39.5%). When queried using NCBI BLAST, the top hit (at 97% identity) was most closely related to an “uncultured bacterial clone” from Antarctic glacial ice (GenBank Accession: KP190124.1). It is not clear whether this chloroplast sequence belongs to a cyanobacterial or eukaryotic species.

### Aufeis samples

Aufeis samples, A1–A6 (top right panel, Figure 1C; see Figure S2 for site locations), which are spread along a roughly 2 km area of the Sulfidic Aufeis, appeared to be layered in place, indicating that multiple flow and freeze cycles had occurred during their deposition. Sulfide was also measured for aufeis samples from sites A1 and A4 and ranged from 0.32 to 0.52 mM, respectively (Table 1). Sulfate values for aufeis sites A1–A4 and A6 ranged in value from 0.07 to 8.62 mM. Chloride concentrations for aufeis sample with associated geochemistry ranged from 0.1105 to 23.32 mM (A6 and A3, respectively).

**Figure 4:**
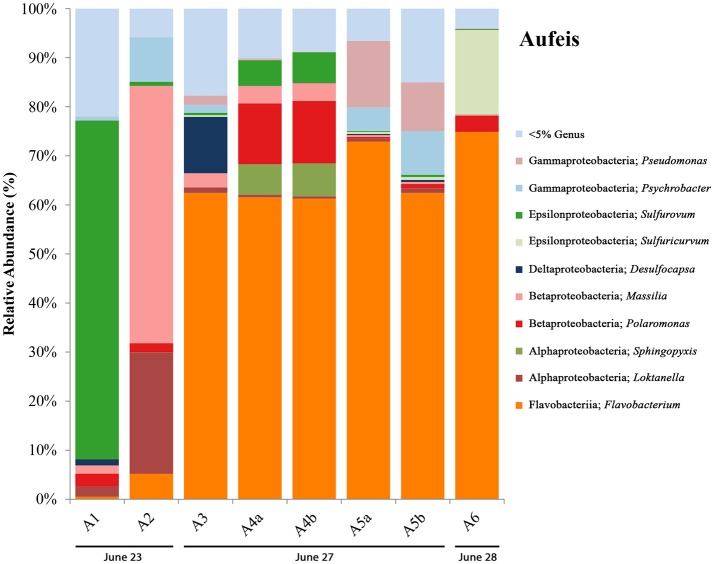
Aufeis bar charts show relative abundance of 16S rRNA gene sequencing libraries, where only OTUs representing organisms more than 5% of any given sample are labeled. All other OTUs below 5% relative abundance are grouped together in the category “Genus <5%.” Labels show Class and subsequent Genus level. Sites are named such that any biological replicates are denoted by lowercase letters. Aufeis samples are in order (from left to right) according to date sampled. Their order as they appear in the Sulfidic Aufeis zone can be seen in Figure S2.

16S rRNA gene sequencing data for aufeis samples were obtained across six different sites with varying distances in relation to the Ice Blister (see Figure 1B; Figure S2). Figure 4 is a bar chart of these sites organized by sampling date from left to right. Their orientation along the Sulfidic Aufeis can be seen in Figure S2. Similar to the melt pools are OTUs most closely related to bacterial genera that were observed in polar environments previously, such as *Pseudomonas, Psychrobacter, Massilia, Polaromonas*, and *Loktanella*. Similarly, we found the presence of the SOM *Sulfurovum*, as well as *Sulfuricurvum*. In previous research, Wright et al. (2013) found these two SOM OTUs to be abundant in sediment samples, typically with *Sulfurovum* being the more abundant of the two organisms. In two of the aufeis sites, *Sulfurovum* represents ~69% of site A1 and ~6% of site A4. However, further down the Sulfidic Aufeis in Site A6 we observed *Sulfuricurvum* representing 17.2% of organisms, whereas *Sulfurovum* is less than 1% present. *Desulfocapsa* was also present in site A3 which is roughly 40 m south of the Ice Blister and the closest of all aufeis sites within the Sulfidic Aufeis zone. OTUs most closely related to the genus *Flavobacterium* were well represented in aufeis site samples. *Flavobacterium* sp. was the most dominant organism present in sites A3, A4, A5, and A6 from ~61% all the way up to 75%. The dominant *Flavobacterium* OTU in melt pool samples was also present in aufeis samples. Another OTU was also present and was most closely related to *Flavobacterium aquidurense* (99%) when queried against the NCBI BLAST database. Additionally, an OTU most closely related to the Alphaproteobacterial genus *Sphingopyxis* represents ~7% in site A4. This organism was previously identified as a marine microorganism with cold-adapted proteins (Ting et al., 2010).

### Mineral precipitate samples

Also littering the Sulfidic Aufeis were piles of white/yellow mineral precipitates (AS1-AS7) that ranged from crystalline to soft, goopy piles of material most likely made up of elemental sulfur, calcite, gypsum, or other sulfur minerals (Grasby, 2003; Grasby et al., 2003; Lau et al., 2017). These samples (bottom left panel, Figure 1C; Figures S3b,c) were often observed in areas that appeared to be older and less active, unlike a melt pool. Two mineral precipitate samples (AS1 & AS2) were collected from 2014 (Figure 1B), while all others (AS3-AS7) were collected during 2017. All 2017 mineral precipitate samples were taken from the area that remained from the 2014 sampling effort (Figure S3a). Because samples AS1 and AS2 were filtered, one (AS2) had enough residual fluid to be submitted for aqueous geochemistry. Sulfate values for AS2 were 3.225 mM, while chloride was measured at 0.2912 mM. No aqueous geochemistry data was captured for 2017 mineral precipitate samples (there was no liquid water).

**Figure 5:**
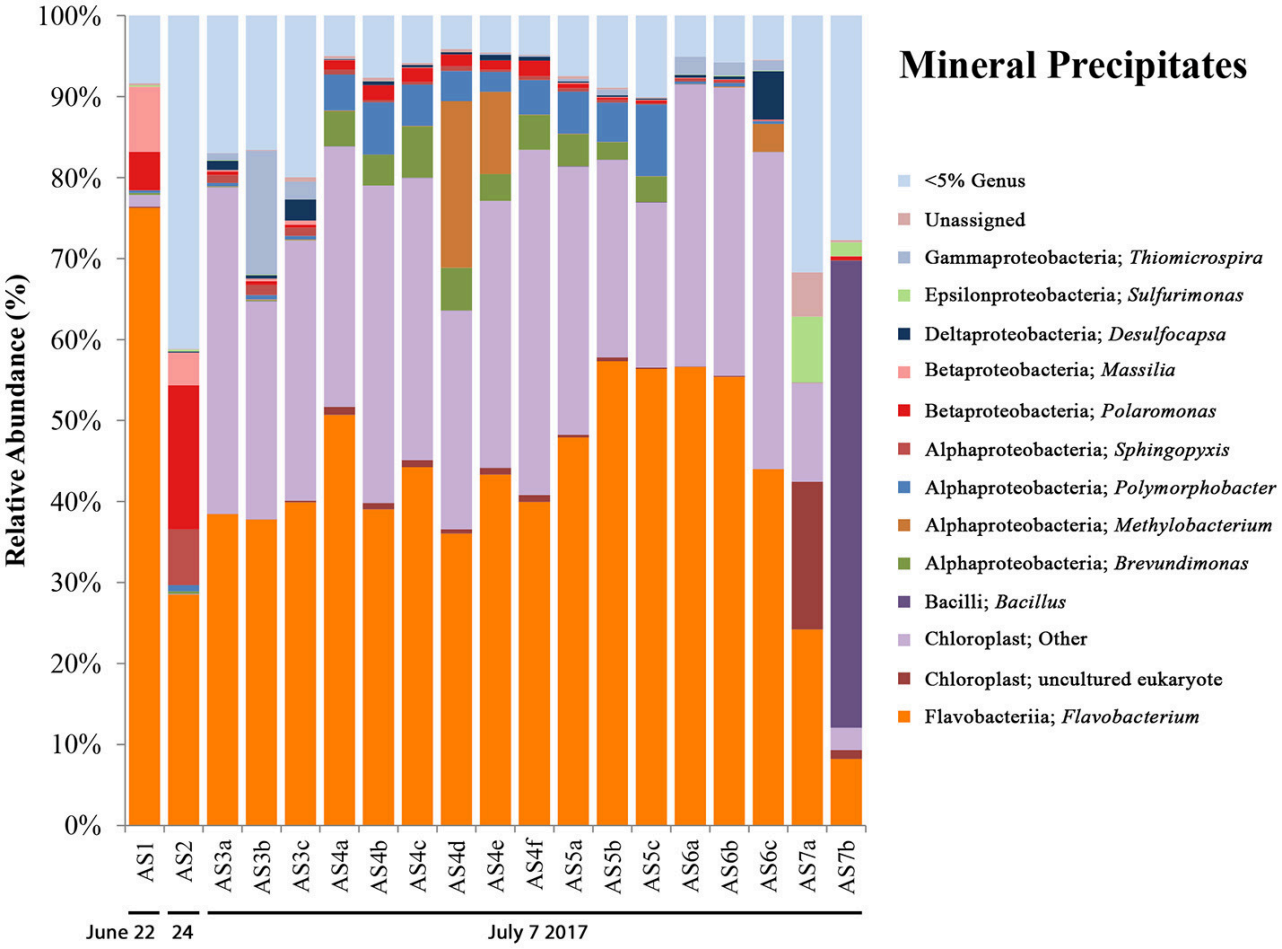
Mineral precipitate bar charts show relative abundance of 16S rRNA gene sequencing libraries, where only OTUs representing organisms more than 5% of any given sample are labeled. All other OTUs below 5% relative abundance are grouped together in the category “Genus <5%.” Labels show Class and subsequent Genus level. Sites are named such that any biological replicates are denoted by lowercase letters. The mineral precipitate sites are in order (from left to right) based on sampling date. Two samples are from 2014 (AS1 & AS2), while the rest are from 2017 (AS3–AS7). The 2017 sampling area is shown in Figure S3.

16S rRNA gene sequencing data for mineral precipitate samples (see Figure 5) includes AS1 and AS2 (from 2014, with one replicate each) and five samples collected during the 2017 field campaign (AS3-AS7, with three biological replicates for each, except for AS7 which had two). The microbial communities observed in the mineral precipitates were also present in melt pool and aufeis sites and included OTUs most closely related to the genera *Massilia, Polaromonas, Sphingopyxis*, and *Brevundimonas*. Present in the 2017 samples were organisms not previously seen at BFP, including OTUs most closely related to the Gammaproteobacterial genus *Thiomicrospira* (sites AS3 and AS6), and the Alphaproteobacterial genera *Polymorphobacter* (sites AS4 and AS5) and *Methylobacterium* (sites AS4 and AS6), all of which were seen in relatively small but consistent amounts (~2, ~5, and ~10% respectively). All three OTUs were previously found in Arctic and Antarctic environments (Niederberger et al., 2009; Christner et al., 2014; Kleinteich et al., 2017). *Sulfurimonas* was present in small relative abundances in site A7, and *Desulfocapsa*, while present in very small amounts in almost all samples was most prevalent in site A6 (~6%). The most abundant OTU most closely related to *Flavobacterium*, comprising approximately 40–50% abundance of all samples except for site AS7b. This is the same OTU present in melt pool sites (genus: *Psychrolimnae*). This site also contained a large abundance of an OTU most closely related to a *Bacillus* species. Also of high relative abundance were OTUs classified as chloroplast, one of which was also present in two of the melt pool samples. These OTUs were highly represented across all 2017 mineral precipitate samples (averaging above 30% abundance); with only a small percentage (~1.5%) present in 2014 sample AS1. The most abundant OTU in mineral precipitate samples, when queried against NCBI BLAST resulted in a 97% identical match with an “uncultured eukaryote clone” (GenBank Accession: EU005289.1).

### Glacier samples

**Figure 6:**
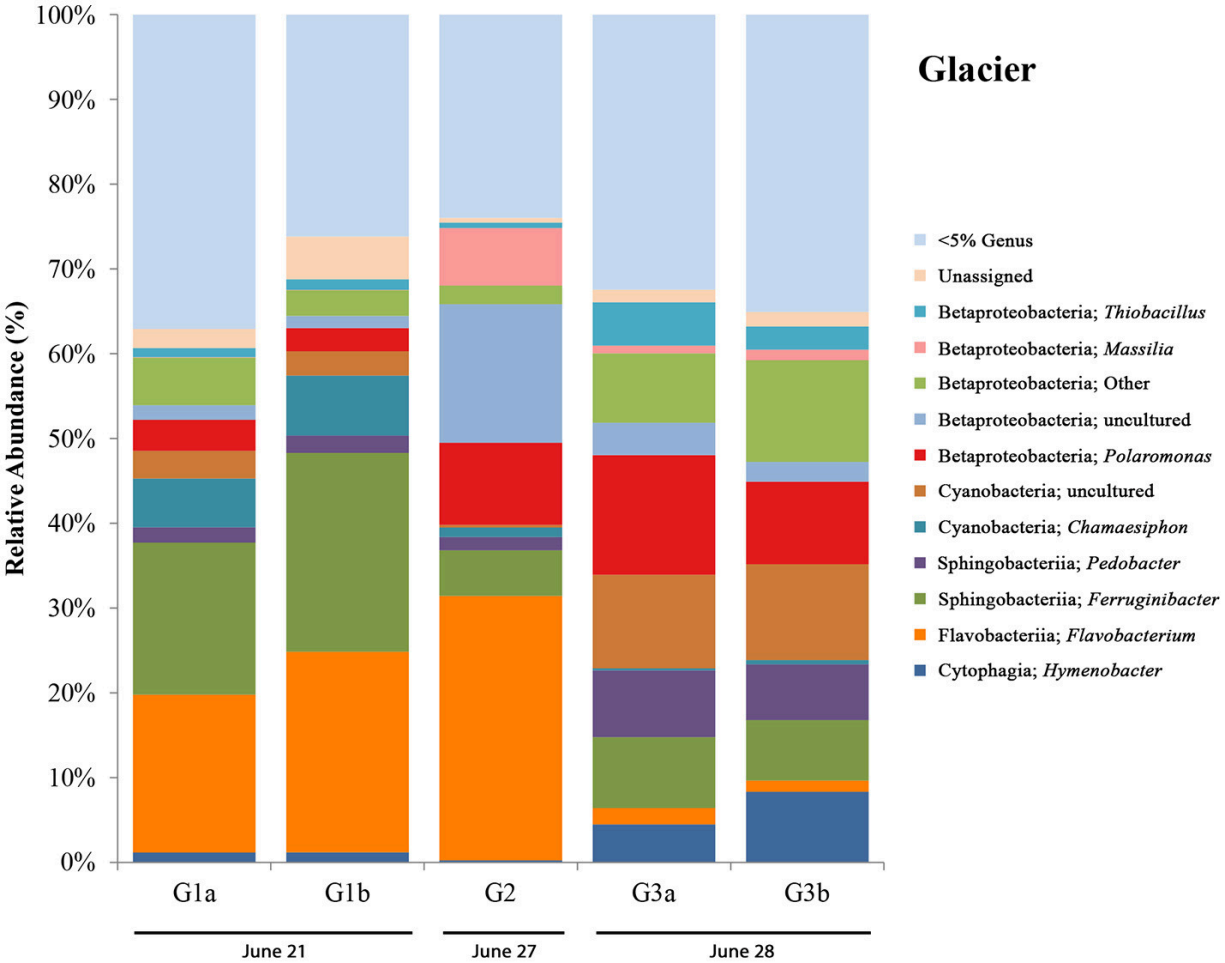
Glacier bar charts show only OTUs that are represented above 5% in any given sample. Sites are ordered (from left to right) according to sampling date. G1-G3 only include those control sites where DNA was extracted for 16S rRNA sequencing. Only site G2 also has aqueous geochemistry data associated with it. Locations for glacier sites can be seen in Figure 1B.

Glacier samples (bottom right panel, Figure 1C) were collected outside of the Sulfidic Aufeis zone to be used as comparisons for aqueous geochemistry (G2, Figure 2 and Figure S4) and 16S rRNA gene sequencing data (G1–G3, Figure 6). The locations of these sites are also displayed in Figure 1B, two of which are north of the Sulfidic Aufeis zone (G1 and G3) and one almost two kilometers to the east of the Sulfidic Aufeis (G2). Site G3 is similar to an aufeis site as it was processed the same way, where subsurface ice was collected and filtered. Site G1 was a melt pool with cryoconite material that was also collected and filtered. Site G2 was ice-melt/glacial runoff, and is the furthest site from the Ice Blister. Sulfate for sample G2 was 0.0146 mM, while chloride was measured at 0.0128 mM. These values are similar to those shown in Figure 2 (Control; values not reported).

16S rRNA gene sequencing data for glacier sites (G1, G2, and G3) are summarized in Figure 6. Surprisingly, in all sites we observed the presence of an OTU most closely related to *Thiobacillus* (with the highest abundance being in G3 at ~5%), a sulfur-cycling microorganism, which we did not expect to see outside of the Sulfidic Aufeis zone. The common polar genera *Massilia* and *Polaromonas* are present in various abundances in all glacier samples, and also seen alongside two Betaproteobacteria OTUs that are classified as “Other” and “uncultured.” An OTU most closely related to the cyanobacterial genus *Chamaesiphon*, which was found previously in Arctic habitats (Lionard et al., 2012), was present and in its greatest abundance (~7%) at site G2, just north of the Ice Blister. This organism was also accompanied by an uncultivated cyanobacterial OTU found in both sites G1 and G3, with its greatest abundance in site G3 at ~11%. Two OTUs most closely related to the genera *Pedobacter* and *Ferruginibacter*, both in the class Sphingobacteriia, were represented in these samples (~3 and ~10% respectively), and were previously found in Arctic environments (Zhou et al., 2012; Peter and Sommaruga, 2016). While OTUs most closely related to the genus *Flavobacterium* were not as abundant as observed in melt pool, aufeis, and mineral precipitate sites, it was still present in relatively large abundances especially in sites G1 and G2 (~20 and ~30% respectively). The most prevalent *Flavobacterium* OTU in these samples was the same as reported in the aufeis (genus: *Aquidurense*). Furthermore, the presence of an OTU most closely related to the genus *Hymenobacter* was seen at ~6% in site G3. This genus was previously isolated from marine sediments in the Arctic (Kim et al., 2017).

### Additional aqueous geochemistry

Aqueous geochemistry data for the 2016 spring, aufeis, melt pools, mineral precipitate, and glacier sites are shown in Table 1. Geochemical data reported for an active spring in 2009 by Wright et al. (2013) is included for comparison. Charge balance error (CBE) was calculated for all sites (Table S3) in order to constrain the accuracy of aqueous geochemical results. The aqueous geochemistry of surveyed sites is characterized by salty, sulfide-rich fluids from both melt pool and aufeis samples, as well as those seen in spring fluids collected from previous years (Grasby et al., 2003; Gleeson et al., 2010; Wright et al., 2013). This indicates that melt pools and aufeis are not directly derived from glacial ice or glacial melt waters, but rather most likely trace back to the subsurface/sub-glacial spring source.

Aqueous geochemistry was explored via a PCoA plot (Figure S4) of BFP sites and their associated ion concentrations. The results reveal that the analytes driving geochemical diversity in the system are Na^+^, Cl^−^, Ca^2+^, and ${\text{SO}}_{4}^{2-}$. Sample sites also appear to follow a trend in sulfate-chloride space (Figure 2). Sodium ion values ranged between below detection (G2) and 39.62 mM (2016 Spring). The highest reported sodium value however belonged to the 2009 Spring at 51.80 mM (Wright et al., 2013). Calcium ion values range from 0.0053 (G2) and 14.7 mM (2016 Spring) and follow the same trend as sulfate values seen in the log-log aqueous geochemical ordination of these two ions (Figure S4). In general, 2009 and 2016 spring samples had the highest total analyte values followed closely by the melt pools and then the aufeis and mineral precipitate samples (AS1 & AS2 from 2014). The glacier sample (similar to “Control” samples) had the lowest overall values for sulfate and chloride and showed an overall negative correlation in the geochemical ordination (Figure S4).

The pH for sites M1 and M2 was 5.5–6.8 and 5.5–6.0, respectively. pH values for aufeis sites and 2014 mineral precipitate sites were not measured. Measured pH for sites AS3 and AS4 (from 2017) was approximately 7 (Table 1). pH measurements were not taken for other sites.

## Discussion

We used 16S rRNA gene sequencing coupled to geochemical analysis to identify and better understand the microbial communities at BFP that have adapted to live in this sulfur-rich, low-temperature environment. This was accomplished over multiple field campaigns to BFP to collect samples from multiple site types (spring discharge, aufeis, mineral precipitates, and melt pool waters). Overall, we interpret the 16S rRNA gene sequencing data presented (Figures 3–6) across multiple site types, to show that BFP is a highly dynamic system over short time periods (weeks to months). While over longer durations (years) a basal microbial community exists and is influenced temporally by sulfur-cycling microorganisms on smaller time scales during freeze/thaw and/or spring flow events. Samples from multiple site/material types were collected in order to evaluate the potential differences/similarities between their associated microbial communities. We have included the 2016 spring 16S rRNA gene sequencing data with the melt pool data due to the presence of similar microorganisms (Figure 3) and fluid geochemistry (Figure 2 and Figure S4) between 2014 melt pool sites and 2016 spring discharge fluid data. Aufeis sites, while different, are similar to melt pool sites in that they are the precursors to melt pools. Both melt pools and aufeis are spring-derived, with melt pools being a more active melt-thaw system derived from static in-place, frozen aufeis. It is important to note that samples for melt pool sites were taken from surface pools, while aufeis samples were from melted ice that was dug from below the surface. It should be noted however, that mixing trends between glacial runoff and melting aufeis are not wholly understood and should be further investigated in order to determine whether or not this could be influencing microbial community dynamics.

A successional microbial community trend appeared within the melt pool and aufeis sites over the course of the 2014 field sampling. One key example of this can be seen in site M3, where M3a was sampled on 26 June, 2014 along with site M2. A large, dominant community of *Sulfurimonas* is seen in these samples. When site M3 was sampled for a second time (M3b; 30 June, 2014), *Sulfurimonas* is far less and *Flavobacterium* is shown to be dominant (Figure 3). This can be seen with aufeis samples as well, as sites A1 and A2 (sampled on 23 June 2014) have a very low abundance of *Flavobacterium*, while sites A3-A6 (all sampled on or after 27 June, 2014) show this as the dominant organism (Figure 4). While the aufeis are more in terms of site composition, the apparent growth of *Flavobacterium* in this case could be due to the speed of melting of the site thus providing a dynamic to the microbial community structure. Aufeis samples were “slushy” and it's possible that increased melt during this time of the season allows a fast proliferation of dominant microorganisms. The addition of 2017 mineral precipitate samples (which are remnant from the Ice Blister in 2014) shows a similar long-term trend suggesting that even over larger timescales (in this case 3 years), these communities, dominated by *Flavobacteria* and an organism with sequences classified as chloroplast (likely Cyanobacteria or glacial algae) can persist (Figure 5). While our data show that different sulfur-oxidizing microorganisms (SOMs) do change over time and sample type/material, there are consistent organisms present (i.e., *Flavobacterium* sp.). The apparent succession of sample types appears to go in order of melt pool/spring fluid to aufeis, and then finally to mineral precipitates. As ices/materials get older the *Flavobacterium* continues to dominate while chloroplast-containing organisms (possibly Cyanobacteria, but most likely glacial algae) take hold as well. This may indicate that more dynamic site types are more amenable to SOMs, whereas more static or stable site types are preferable for *Flavobacterium*.

*Flavobacterium* is not new to BFP as reported previously (Wright et al., 2013). Wright et al. (2013) suggested that in association with SOMs, *Flavobacterium*, which is an aerobic organism, may be creating a “microoxic” environment in surface sediments where SOMs were able to thrive at lower O_2_ levels (Inagaki et al., 2003, 2004). Furthermore, it has been suggested that *Flavobacterium* may be utilizing organic carbon present at the site via the degradation of complex molecules. It is not known however, if the detected dissolved organic carbon (DOC) previously found at BFP (3.9–11.4 mg L^−1^; Wright et al., 2013) is delivered from subsurface organic-rich shales to the surface via spring fluids directly or is being formed *in situ* via microbial metabolisms and/or being atmospherically delivered. Boetius et al. (2015) reported that many Arctic and Antarctic sea ice communities were found to be dominated by the bacterial classes Flavobacteriia and Gammaproteobacteria, mainly due to their ability to breakdown DOC and extracellular polymeric substances (EPS) produced by sea ice algae. It must be noted that the *Flavobacteria* at BFP are commonly found in conjunction with chloroplast-containing and cyanobacterial organisms (site M4, M5, M6, all mineral precipitate sites, and G1 and G2, respectively). These findings suggest that *Flavobacterium* at BFP may not actually be contributing directly to any type of sulfur cycling; rather they are merely part of the basal microbial community that inhabits the surrounding area. *Flavobacterium* species are ubiquitous on the planet and found in both aquatic and terrestrial environments with over 100 species identified (Kolton et al., 2013). Moreover, they have also been found to be associated with snowfall events in subalpine environments (Honeyman et al., 2018). It is likely in this case that *Flavobacterium* found at BFP are niche species that have adapted to live in a sulfur-rich, sub-aerial environment, and are possibly acting to breakdown complex organics to be used by other microbial community members [e.g., sulfate-reducing microorganisms (SRMs)].

*Desulfocapsa* was found in a majority of samples including 2016 spring, melt pool, aufeis, and mineral precipitate sites, and its presence throughout BFP samples is further evidence that the reduction of some oxidized forms of sulfur (e.g., sulfite, etc.) is at least partially microbially mediated at BFP. Previous sulfur isotopic data has been interpreted to show that sulfide delivered from the subsurface is produced via biological sulfate reduction (BSR; Grasby et al., 2003, 2012; Gleeson et al., 2012). While the organisms potentially driving such BSR have not yet been identified in unobtainable subsurface/subglacial samples, perhaps the genus *Desulfocapsa* present in BFP samples represents a fraction of subsurface microbiota. While *Desulfocapsa* is actually found in at least one sample from each site type, the majority of its presence is within melt pool sites. This further strengthens the concept that BFP Spring fluid is closely related to melt pool sites and may even actively seed surface processes via subsurface microbial communities. This is the first time that microorganisms related to sulfate reducing bacteria have been found at BFP. Moreover, the presence of organic carbon (even in a small amount of 0.20 wt.%; Lau et al., 2017) within M2 (where *Desulfocapsa* is present) helps to support this claim.

*Desulfocapsa* sp. have been implicated previously in the disproportionation of inorganic sulfur compounds (Janssen et al., 1996), including elemental sulfur and thiosulfate (Finster et al., 1998). More recently it has been found that *Desulfocapsa sulfexigens* specializes specifically in the disproportionation of elemental sulfur, thiosulfate, and sulfite, using CO_2_ as its sole carbon source (Finster et al., 2013), and even more intriguingly, is unable to grow on sulfate reduction. This may suggest then that the large presence of S^0^ at BFP, especially around the Ice Blister may partially be metabolized by *Desulfocapsa*. Furthermore, this may help to explain its presence in 2017 mineral precipitate samples (Figure 5) where samples were collected from brightly yellow materials. While 16S rRNA gene sequencing reads from BFP are not resolved enough to tell us the species of *Desulfocapsa* that are present, it may be possible that disproportionation of these inorganic sulfur species is occurring *in-situ*. This disproportionation process could thus be responsible for the kinds of sulfur found to be present on-site (e.g., α-, β-, γ-cyclooctasulfur; Lau et al., 2017), however, it has not been determined whether or not these rare allotropes are biogenic in origin. Moreover, it is not clear how a putatively anaerobic microorganism is actively metabolizing in a surface, oxygenic environment.

Because of the expected low biomass in the system we used extracted DNA concentrations to approximate the quantities of microbial cells in the BFP system by site type (Table S3). It is challenging to extract DNA from these types of systems where nucleic acids tend to adsorb strongly to mineral-rich samples (Direito et al., 2012). Lower sequencing depth is not unexpected, especially for environmental samples and low biomass environments (Boetius et al., 2015; Stibal et al., 2015). While site values fluctuate (e.g., 4.95 × 10^2^ − 9.80 × 10^3^ cells mL^−1^ or cells mg^−1^ in melt pools), there are minimal differences between melt pools and aufeis sites, which both have averages between 2 and 4 × 10^3^ cells mL^−1^ or cells mg^−1^. The only noticeable difference is seen in the 2017 mineral precipitate sites that averaged 1.55 × 10^5^ cells mL^−1^; however, this may be attributed to better chemistry in the ZymoBIOMICS™ DNA/RNA Mini Kit (Zymo Research Corp.). This is further evidenced in the 16S rRNA gene sequencing data where the average number of sequence reads for melt pool sites was 7,541 reads, and 6,172 for mineral precipitate sites. While there may have been a difference in extraction kit chemistry, the average values for sequencing reads is close and doesn't affect the interpretation of the data.

Aqueous geochemistry across site locations follows a clear trend of higher ion concentrations found in spring fluids, followed by melt pools, aufeis, and finally glacier samples. This logically makes sense in that melt pool sites are surface level, and most likely the most recent form of aufeis. Aufeis samples, as noted in the methods and materials, are thought to be older ice, presumably layered, and possibly diluted slightly from any glacial melting or meteoric input. Previous aqueous geochemistry from the 2009 BFP spring (Wright et al., 2013) trends very well with data collected from two melt pool sites (M2 and M3) as well as spring fluid collected during 2016 (Table 1). The ordination of aqueous geochemical data (Figure S4) shows a strong correlation of the 2009 and 2016 spring fluids and saline waters. This correlation is pronounced with melt pool samples M2 and M3 as well as aufeis sample A3. The remaining samples show a negative correlation in the ordination. Graphing ions against each other (e.g., ${\text{SO}}_{4}^{2-}$ and Cl^−^) is a common geochemical method to discover whether or not certain analytes are trending together. The log-log plot (Figure 2) generated for this purpose supports the same findings from the geochemical ordination in Figure S4. Sulfate and chloride increase in concentration together and those associated sites are seen among melt pools, aufeis, and the 2009 and 2016 spring fluids. Due to the remote location of BFP and works done to date, knowledge of the underlying hydrology of the glacier is poorly understood. However, it is possible that variability in ion concentrations, especially in spring geochemistry is governed by stochastic flow of fluids in the subsurface. Moreover, as these fluids contact the surface and begin mixing with glacial melt we would also expect more variability in ion concentrations between the different site types as well.

When comparing aqueous geochemistry to the 16S rRNA gene sequencing data there doesn't appear to be any general trends. Of the sites with the highest total dissolved solids, sites M2 and M3 both have high relative abundances of the OTU most closely related to *Sulfurimonas*; however, site A3 only has small abundances of the SOMs *Sulfurovum* and *Sulfuricurvum*. Furthermore, the 2016 spring fluid and site A3 have high abundances of *Flavobacterium*, which strangely enough is not the case for 16S rRNA gene sequencing data for the 2009 Spring where an OTU most closely related to *Burkholderia* was the dominant organism (Wright et al., 2013). The 2016 spring also has a large abundance of *Sulfurovum*, which is also seen as the primary SOM in site A1. The only SOM seen in abundance in mineral precipitate samples is *Sulfurimonas* in site AS7 which is an ice scraping seen in Figure S3b. There isn't any clear reason why these SOMs are in different abundances in different samples at different times, but it may very well have to do with acute events that select for given metabolisms allowing one to flourish when others do not. How aqueous geochemistry interplays with sulfur-cycling organisms at these sites remains uncertain as 16S rRNA gene sequencing only provides us with taxonomic classification, and we can only infer potential metabolic functionality. On-going metagenomic work will shed light on these questions.

While we do present sulfide data for a few of the included sites, it remains unknown how sulfide plays a role in the system other than potentially being used by SOMs as an energy source. The link between sulfate and chloride concentration may indicate that some of the microorganisms present, possibly the SOMs, have a higher tolerance to these increased ion concentrations. However, there is not a clear distinction between sites with high relative abundances of SOMs and those without where ion concentrations are high. In spring discharge fluids, melt pools, and aufeis sites, there are multiple samples where either SOMs have the highest relative abundance or *Flavobacterium* has the highest relative abundance. There is no clear delineation as to whether one set of organisms is more tolerant than the other. What is clear is that *Flavobacterium* can live at high and low concentrations of sulfide, sulfate, and chloride as evidenced in its presence in all samples along that geochemical gradient.

Microorganisms that can survive and even thrive in extreme environments on Earth have been of great interest in the study of astrobiology. One way to inform upon the types of organisms and their interactions within an environment beyond Earth is to study terrestrial environments well suited as potential analogs for extraterrestrial bodies. The Galilean moon Europa is a target for current astrobiological investigations due to the presence of a subsurface, liquid-water ocean (Kivelson, 1997; Carr et al., 1998), as well as hydrated sulfate salts and sulfuric acid that have been detected in deposits on the icy surface (Carlson et al., 1999; McCord et al., 1999), with the ocean potentially capable of supporting life (Hand et al., 2007; Cockell et al., 2016). The presence of surface-deposited sulfate salts and sulfuric acid supports the hypothesis that sulfur-based metabolisms could be fueled by water-rock interactions within and/or below the ocean, coupled with the delivery of oxidants via the cycling of surface ice (McCollom, 1999; Hand et al., 2007).

BFP presents a system where subsurface fluids emerge at the toe of an Arctic glacier, sometimes even through conduits formed within the glacial ice itself. These supraglacial spring fluids are then introduced to a much different surface environment. For this reason, BFP has been proposed as one of the key analogs for Europa on Earth (along with other spring sites such as Blood Falls and regions where ice sheets separate surface environments from cold ocean waters below). Although the surface environment at BFP does not directly reflect the surface environment at Europa (approximately −170°C, ~10^−8^ torr, highly ionizing radiation, etc.; McCord et al., 2002; Hand and Carlson, 2015), the potential for interchange between Europa's ocean and the surface via the cracks within the icy crust (the lineae) as well as the abundance of sulfur-bearing compounds on Europa (Carlson et al., 1999; McCord et al., 1999) have led to the characterization of BFP as a key analog for Europa. This study has looked at multiple site/material types and the potential changes between microbial communities among them. This is important as glacial systems such as BFP are dynamic in nature where changes to ice and surface material can occur over a relatively short time scale (weeks), but also over extended time scales as well. There is also evidence of a dynamic system on Europa of “chaos terrain” where it appears that the surface has been disrupted from below (Schmidt et al., 2011). Exploring these constantly changing environments on Earth provides a good target for potential sites in the search for life on worlds such as Europa. It may be that the potential for finding life on Europa not only involves searching for organisms that utilize sulfur metabolisms (SOMs and SRMs), but also organisms that are capable of breaking down complex organic matter such as some genera of Gammaproteobacteria and Flavobacteriia (Boetius et al., 2015).

While we attempted to make our sampling efforts as exhaustive as possible, there are always limitations to what can be accomplished in the field. It should be noted that BFP is in a very remote location of the Canadian Arctic, and opportunities to sample there are limited and difficult. Due to field conditions, it was often times difficult to get instruments calibrated and this hindered our ability to collect certain types of data. While some sites had acceptable data for both 16S rRNA gene sequencing and aqueous geochemistry, others did not, hence the difference in biological replicates. One example is our lack of sulfide data which may have provided a better lens through which we could judge the differentiation of SOMs *in-situ*. However, the 16S rRNA gene sequencing data still tells an impactful story about the composition of microbial communities between different glacial sites/materials over time. This, along with aqueous geochemistry data, have allowed us to further enhance our knowledge of microbial organisms inhabiting these low-temperature sulfur systems on Earth and possibly extraterrestrial worlds such as Europa. This information may be valuable in the preparation for sampling by a Europan lander as a difference in material type (melting and filtering ice vs. preserving mineral precipitate scrapings) may require a difference in approach based on the potential microbial communities present. Moreover, based on how dynamic glacial environments can be on Earth, we recommend that a Europan lander focus on similar sites such as the “chaos terrain” discussed by Schmidt et al. (2011).

## Conclusion

This study used 16S rRNA gene sequencing coupled to geochemical analysis to better constrain the microbial consortia that have adapted to live in the sulfur-rich, low-temperature environment at BFP. We successfully identified new dynamics of microbial communities present at BFP, namely, how microbial assemblages are dispersed between different site/material types and locations, and which communities tend to persist over long and short-term time periods. Our results demonstrate the widespread presence of sulfur cycling organisms (i.e., *Sulfurovum, Sulfuricurvum, Sulfurimonas*, and *Desulfocapsa*) across these site types and in varying relative abundances. Results also show that a baseline microbial community exists within most samples regardless of sample type, typically consisting of a large relative abundance of an OTU most closely related to the genus *Flavobacterium*. This suggests that much like “chaos terrain” found on Europa, dynamic environments, whether spring fluid, melted ice, or even salt-like scrapings may contain similar microbial communities, and therefore be ideal targets for astrobiological investigation. Moreover, while the focus in previous studies has been on traditional sulfur-cycling microorganisms, our data points to a potentially larger role of *Flavobacterium* sp. based on their ubiquity in BFP samples and their persistence over time. Even if they aren't directly contributing to overall sulfur-cycling they may be breaking down large organic molecules for use by other microorganisms (e.g., SRMs). While it is still unclear how the metabolism of *Flavobacterium* sp. influences the environment and geochemistry of BFP, metagenomic and metatranscriptomic research that is underway will shed light on these processes. For example, these data will further elucidate which of the sulfur-cycling microorganisms (*Sulfurimonas, Sulfurovum, Sulfuricurvum*, and *Desulfocapsa*) are actively utilizing sulfur constituents, and in which part of the process they are participating or are most active.

## Data availability

After sequencing, raw reads were deposited into the NCBI sequencing read archive (SRA, https://www.ncbi.nlm.nih.gov/sra) under the accession number SRP136466. The mapping file for samples and corresponding barcodes can be found in Table S1.

## Author contributions

AT, JS, and SG led the design of the study. Fieldwork was conducted by all authors, and laboratory work was done by CT and GL. All authors interpreted results. CT is the primary author of the manuscript with contributions and guidance from all other authors.

### Conflict of interest statement

The authors declare that the research was conducted in the absence of any commercial or financial relationships that could be construed as a potential conflict of interest.
