# Supplementary material for: Low-Temperature Sulfidic-Ice Microbial Communities, Borup Fiord Pass, Canadian High Arctic
Source: Front Microbiol. 2018 Jul 24;9:1622. doi: 10.3389/fmicb.2018.01622 (PMC6066561; doi:10.3389/fmicb.2018.01622)
Supplement: Supplementary file 3 [file Data_Sheet_1.DOCX]

Supplementary Material

Low-Temperature Sulfidic-Ice Microbial Communities, Borup Fiord Pass, Canadian High Arctic

**Christopher B. Trivedi, Graham E. Lau, Stephen E. Grasby, Alexis S. Templeton, John R. Spear***

*** Correspondence:** John R. Spear: [jspear@mines.edu](mailto:jspear@mines.edu)

**Table S1. Mapping file for samples and corresponding barcodes.** This is included as a Microsoft Excel file.

**Table S2. Sequencing coverage summary.** Output of ‘biom summarize-table’ script which gives count statistics for the input BIOM file, including sequencing read numbers, min, max, median, etc. This is included as a Microsoft Excel file.

**Table S3. Sample information table including sequencing counts, charge balance error, and calculated biomass.** Site names are shown with included replicates (a, b, c, etc.). Samples are grouped based on sample type category (A - Aufies; AS - Mineral precipitates; M - Melt Pool; G - Glacier) with included approximate charge balance error (CBE) shown. Biomass values were calculated using DNA concentration and initial sample amount (mass or volume).

| **Sample** | **Date** | **Site Type** | **CBE** | **Seq. read #** | **Sample amount** | **DNA conc. (ng/μl)** | **Biomass (cells/ml)** | |
| --- | --- | --- | --- | --- | --- | --- | --- | --- |
| A1 | 23-Jun-14 | Aufeis | <15% | 6209 | 7570 ml | BD | ND |  |
| A2 | 23-Jun-14 |  | <15% | 12922 | 5678 ml | 0.425 | 1.15E+03 | cells/ml |
| A3 | 27-Jun-14 |  | <15% | 3269 | 5678 ml | BD | ND |  |
| A4a | 27-Jun-14 |  | <5% | 13564 | 2840 ml | 0.828 | 4.46E+03 | cells/ml |
| A4b | 27-Jun-14 |  | <5% | 19106 | 2840 ml | 0.837 | 4.51E+03 | cells/ml |
| A5a | 27-Jun-14 |  | >15% | 5936 | 3785 ml | 0.517 | 2.09E+03 | cells/ml |
| A5b | 27-Jun-14 |  | >15% | 14203 | 3785 ml | 0.318 | 1.29E+03 | cells/ml |
| A6 | 28-Jun-14 |  | <15% | 5615 | Unknown | 4.0 | ND |  |
| AS1 | 22-Jun-14 | Mineral Precip. | >15% | 1243 | 100 ml | 0.4 | 6.14E+04 | cells/ml |
| AS2 | 24-Jun-14 |  | <15% | 10031 | 100 ml | 0.43 | 6.58E+04 | cells/ml |
| AS3a | 7-Jul-17 |  | ND | 8493 | 500 mg | 6.88 | 2.11E+05 | cells/mg |
| AS3b | 7-Jul-17 |  | ND | 11095 | 500 mg | 11 | 3.37E+05 | cells/mg |
| AS3c | 7-Jul-17 |  | ND | 5592 | 500 mg | 2.73 | 8.35E+04 | cells/mg |
| AS4a | 7-Jul-17 |  | ND | 5965 | 500 mg | 7.34 | 2.25E+05 | cells/mg |
| AS4b | 7-Jul-17 |  | ND | 5246 | 500 mg | 5.37 | 1.64E+05 | cells/mg |
| AS4c | 7-Jul-17 |  | ND | 7422 | 500 mg | 6.76 | 2.07E+05 | cells/mg |
| AS4d | 7-Jul-17 |  | ND | 6980 | 500 mg | 3.56 | 1.09E+05 | cells/mg |
| AS4e | 7-Jul-17 |  | ND | 1468 | 500 mg | 4.03 | 1.23E+05 | cells/mg |
| AS4f | 7-Jul-17 |  | ND | 4420 | 500 mg | 0.117 | 3.58E+03 | cells/mg |
| AS5a | 7-Jul-17 |  | ND | 1474 | 500 mg | 4.86 | 1.49E+05 | cells/mg |
| AS5b | 7-Jul-17 |  | ND | 5130 | 500 mg | 5.4 | 1.65E+05 | cells/mg |
| AS5c | 7-Jul-17 |  | ND | 9243 | 500 mg | 5.89 | 1.80E+05 | cells/mg |
| AS6a | 7-Jul-17 |  | ND | 8376 | 500 mg | 4.34 | 1.33E+05 | cells/mg |
| AS6b | 7-Jul-17 |  | ND | 1769 | 500 mg | 9.14 | 2.80E+05 | cells/mg |
| AS6c | 7-Jul-17 |  | ND | 9201 | 500 mg | 3.21 | 9.82E+04 | cells/mg |
| AS7a | 7-Jul-17 |  | ND | 2821 | 500 mg | BD | ND |  |
| AS7b | 7-Jul-17 |  | ND | 10235 | 500 mg | 0.141 | 4.31E+03 | cells/mg |
| G1a | 21-Jun-14 | Glacier | ND | 17103 | 500 mg | 3.91 | 1.20E+05 | cells/mg |
| G1b | 21-Jun-14 |  | ND | 10156 | 500 mg | 1.08 | 3.30E+04 | cells/mg |
| G2 | 27-Jun-14 |  | ND | 4798 | 360 ml | 0.275 | 1.17E+04 | cells/ml |
| G3a | 28-Jun-14 |  | <5% | 2997 | 360 ml | 0.26 | 1.11E+04 | cells/ml |
| G3b | 28-Jun-14 |  | <5% | 11377 | 360 ml | 0.375 | 1.59E+04 | cells/ml |
| M1 | 21-Jun-14 | Melt Pool | <5% | 5177 | 300 ml | 0.0097 | 4.95E+02 | cells/ml |
| M2a | 21-Jun-14 |  | <15% | 13889 | 500 mg | 0.3201 | 9.80E+03 | cells/mg |
| M2b | 21-Jun-14 |  | <15% | 13681 | 500 mg | 0.1679 | 5.14E+03 | cells/mg |
| M2c | 21-Jun-14 |  | <15% | 6032 | 360 ml | BD | ND |  |
| M2d | 21-Jun-14 |  | <15% | 7510 | 500 mg | 0.0401 | 1.23E+03 | cells/mg |
| M3a | 26-Jun-14 |  | <15% | 6007 | 500 mg | 0.0394 | 1.21E+03 | cells/mg |
| M3b | 30-Jun-14 |  | <15% | 4794 | 360 ml | BD | ND |  |
| M4a | 27-Jun-14 |  | <15% | 2478 | 360 ml | 0.0224 | 9.52E+02 | cells/ml |
| M4b | 27-Jun-14 |  | <15% | 10240 | 360 ml | 0.074 | 3.15E+03 | cells/ml |
| M4c | 27-Jun-14 |  | <15% | 7648 | 500 mg | BD | ND |  |
| M5 | 30-Jun-14 |  | <15% | 8242 | 360 ml | 0.179 | 7.61E+03 | cells/ml |
| M6 | 30-Jun-14 |  | >15% | 4795 | 240 ml | BD | ND |  |
| 2016 Spring | 4-Jul-16 | Spring | <15% | 18139 | 2000 ml | 0.406 | 3.11E+03 | cells/ml |


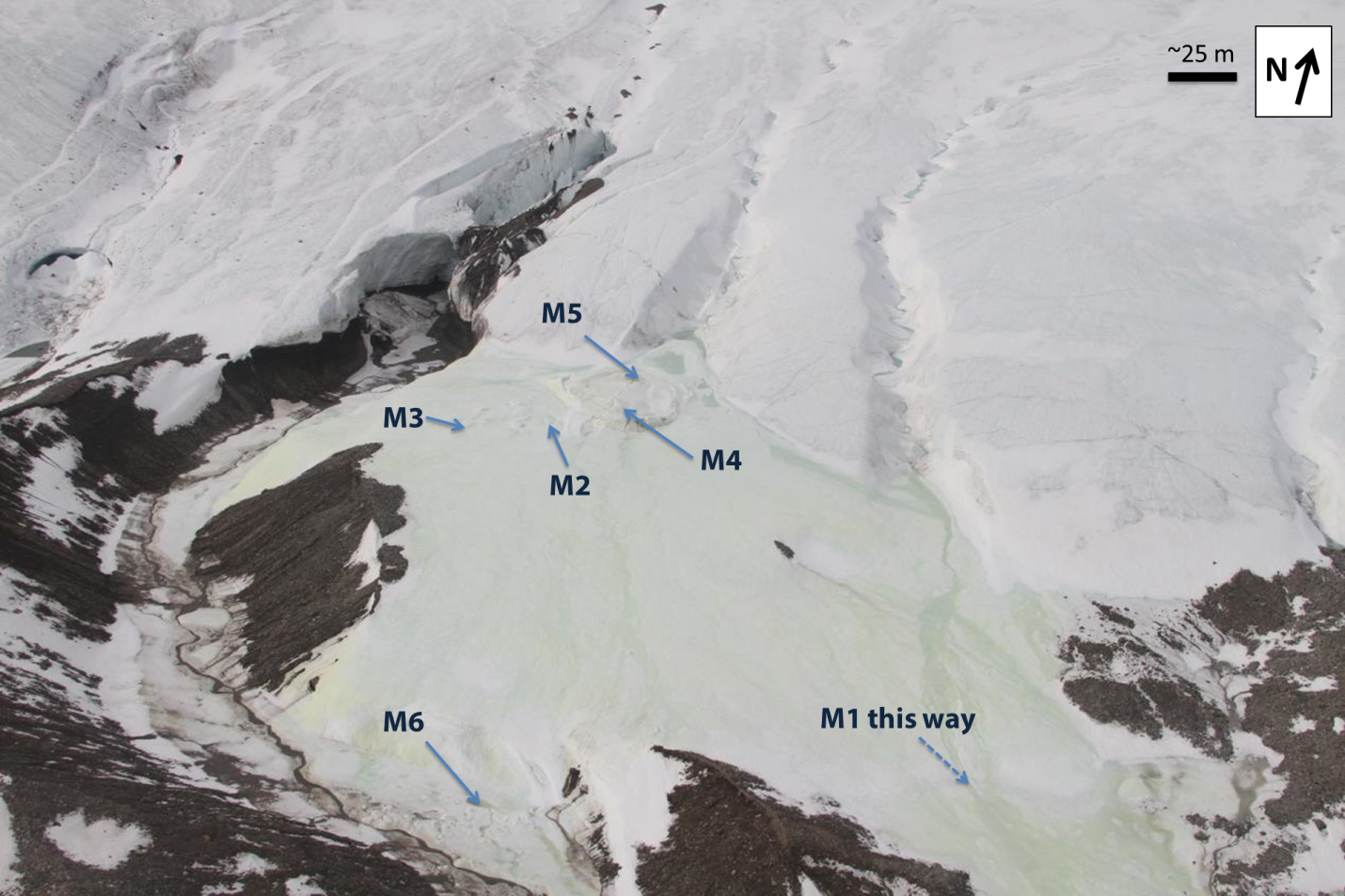


**Figure S1. Melt Pool site locations.** Melt pool locations M2-M6 are shown using light blue arrows. The location of M1 is further down the Sulfidic Aufeis and not shown in this figure, but rather Figure S2. An indicator of the direction of M1 is included. Note the location of sites M4 and M5 directly on top of the Ice Blister (not explicitly highlighted).


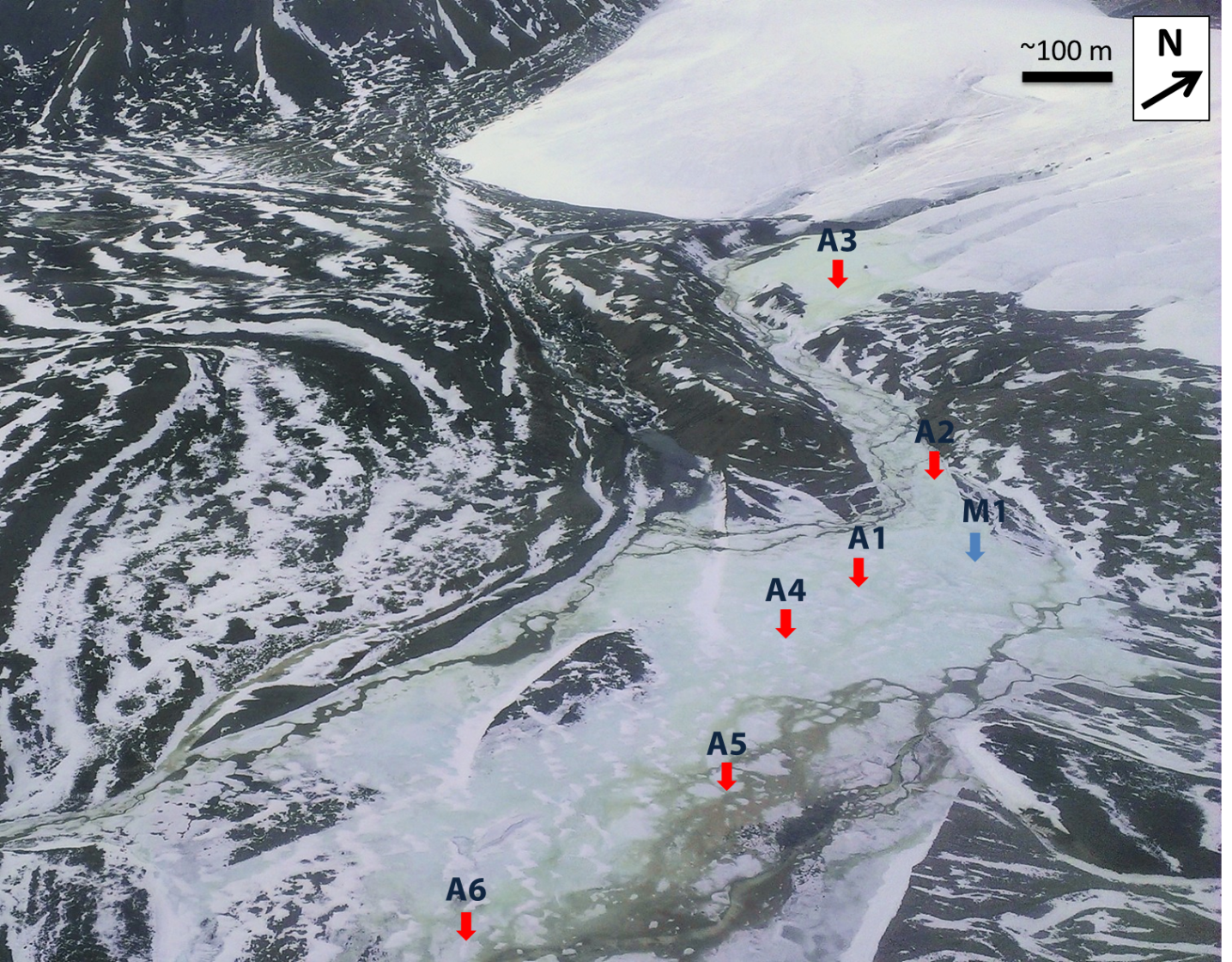


**Figure S2. Aufeis site locations.** Aufeis site locations are shown as they continue down the Sulfidic Aufeis zone. Note the location of M1 which is farther down the aufeis and not able to be shown in Figure S1. Site A3 is the closest to the location of the Ice Blister and the majority of melt pool sites seen in Figure 1b and Figure S1.


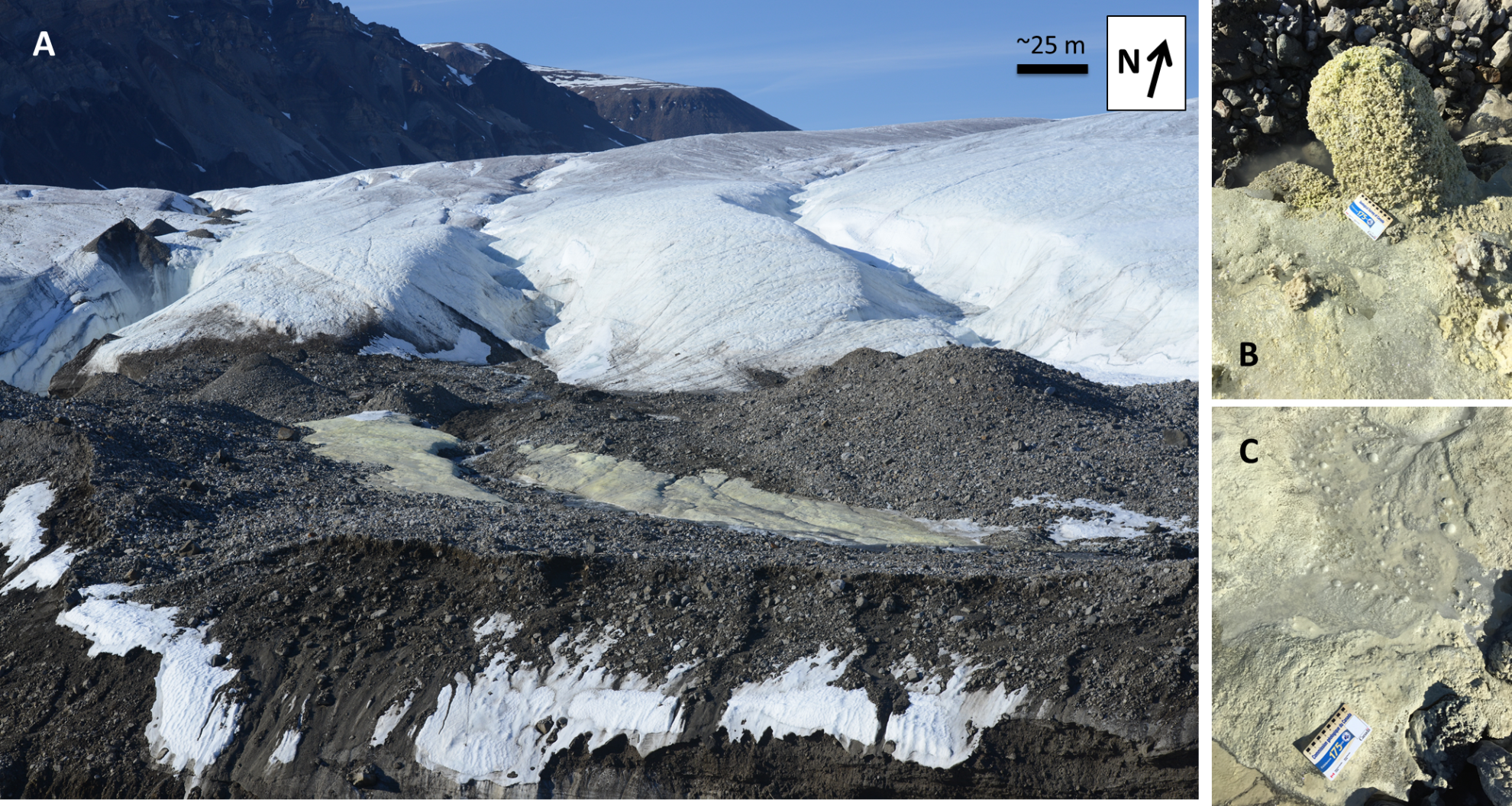


**Figure S3. BFP17 Site.** The general location of the BFP 2014 site as seen on July 7, 2017 (**A**). The yellow staining in **A** is the approximate site of the Ice Blister from 2014. Panels **B** and **C** show examples of samples collected from 2017 and represented as mineral precipitate samples for comparison to 2014 data. The scale bar in panels **B** and **C** is 7 cm, and approximately 8 cm for the total scale card.


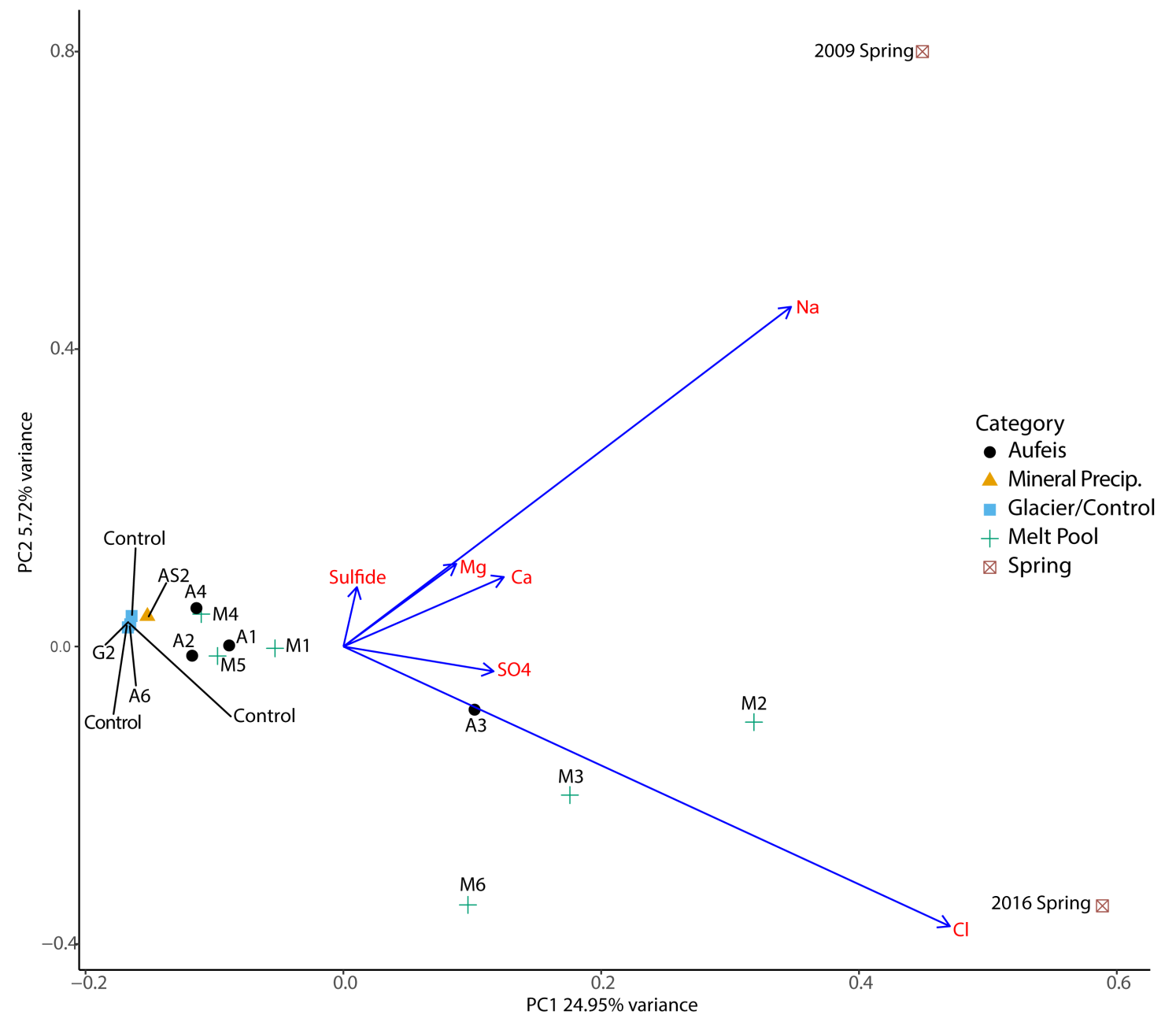


**Figure S4. Principle Components Analysis (PCoA) of BFP aqueous geochemistry.** Samples are separated into categories and ordinated according to geochemical values. The longer the eigenvector (blue arrow) for each value the more strongly it is having an effect on the shown diversity of the system. Sites that are in the same direction of a given eigenvector are considered strongly correlated with that parameter and vice versa for those sites opposite of a given eigenvector. Analytes with eigenvectors of 0 were removed for clarity of the figure. Glacier/Control samples are denoted by the same blue square. Sample G2 has associated 16S rRNA gene sequencing data whereas “Control” samples were used only to compare aqueous geochemistry of the system.


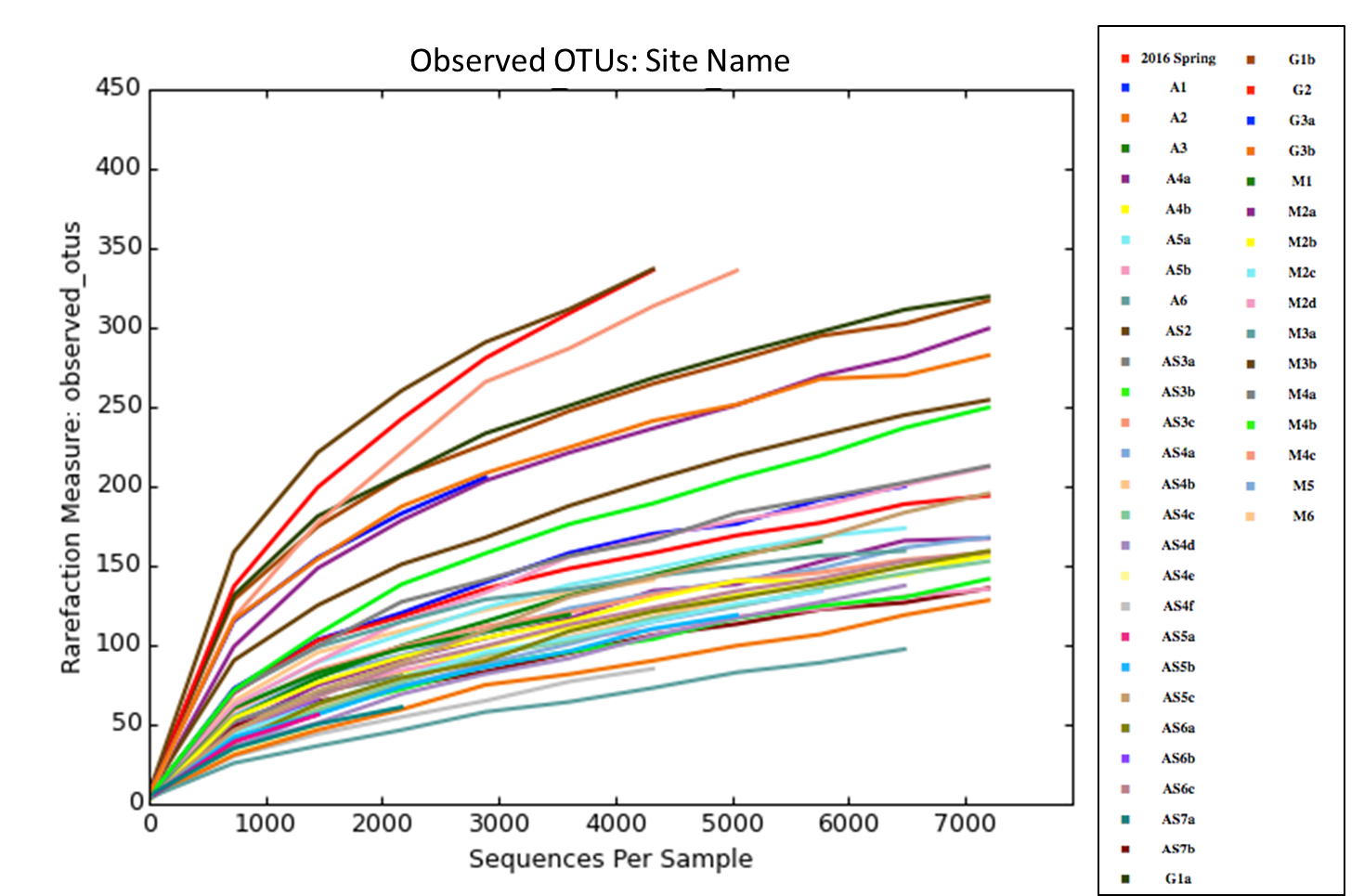


**Figure S5. Alpha rarefaction plot.** Alpha rarefaction plot is shown based on the number of observed OTUs per sample. The upper limit of rarefaction is the median sequence value (6962) which can also be seen in Table S3.


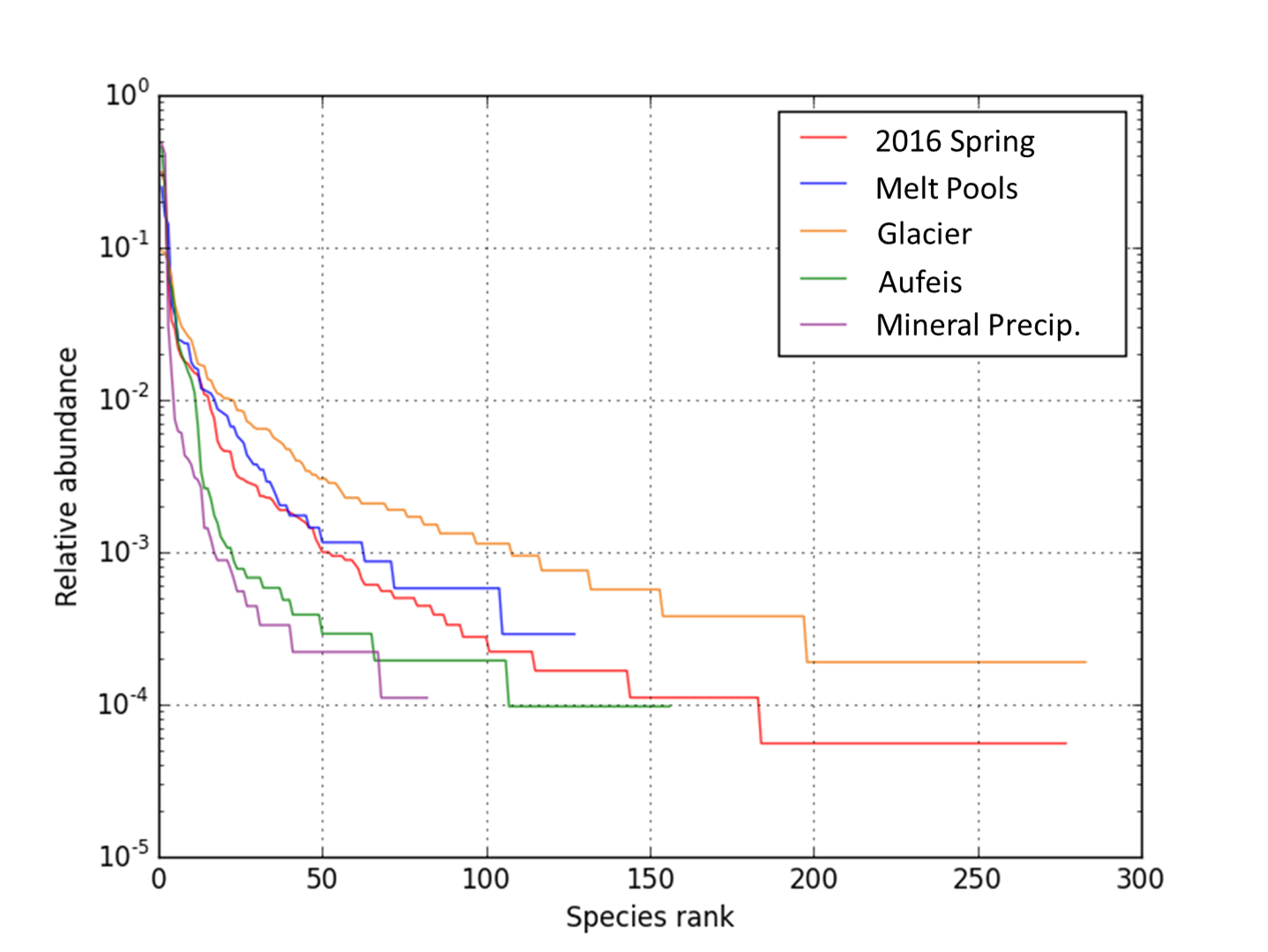


**Figure S6. Rank abundance plot.** Rank abundance is shown by sample type. The y-axis is in log form whereas the x-axis is not. A rank abundance plot is used to show similar diversity across site types in terms of relative abundance vs the number of species (OTUs) averaged across the samples for that site type.
